# Supplementary material for: Amyloid pathology disrupts gliotransmitter release in astrocytes
Source: PLoS Comput Biol. 2022 Aug 1;18(8):e1010334. doi: 10.1371/journal.pcbi.1010334 (PMC9371304; doi:10.1371/journal.pcbi.1010334)
Supplement: S1 Appendix — (DOCX) [file pcbi.1010334.s001.docx]

**S1 Appendix.** Details of the model

**The governing equations of Ca^2+^ dynamics and IP_3_R signaling are below**

d([Ca_cyt_])/dt = *J_PMCA_* + *J_CytB_* – *J_SERCA_* + *J_ER_-_leak_* + *J_IP3R_* + *J_Syt_* 1

d([Ca_ER_])/dt = *J_ERB_* + (*J_SERCA_* - *J_ER_-_leak_* - *J_IP3R_*) *V_Cyt_*/*V_ER_*  2

d([IP_3_])/dt = *J_mGluR_* – *J_3K_* – *J_5P_* + *J_Pδ_* 3

**Ca^2+^ fluxes**

PMCA calcium flux (J_PMCA_) [1] 4

*J_PMCA_* = – *M_0_*[Ca_cyt_]*k_f1_* + *M_1_k_b_* – *M_0_k_l_* 4.1

d(*M_0_*)/dt = – *M_0_*[Ca_cyt_]*k_f1_* + *M_1_k_b_* + *M_2_k_f3_* 4.2

d(*M_1_*)/dt = – *M_1_k_b_* + *M_0_*[Ca_cyt_]*k_f1_* – *M_1_k_f2_* 4.3

d(*M_2_*)/dt = – *M_2_k_f3_* + *M_1_k_f2_* 4.4

Cytosol Ca^2+^ buffer (J_CytB_) [2] 5

*J_CytB_* = – *C_0_*[Ca_cyt_]*k_f_* + *C_1_k_b_* 5.1

d(*C_0_*)/dt = – *C_0_*[Ca_cyt_]*k_f_* + *C_1_k_b_* 5.2

d(*C_1_*)/dt = *C_0_*[Ca_cyt_]*k_f_* – *C_1_k_b_* 5.3

SERCA (J_SERCA_) [3] 6

*J_SERCA_ = V_SERCA_* Hill([Ca_cyt_]^2^, *k_a_*) 6.1

ER-leak (J_ER-leak_) [4] 7

*J_ER-Leak_* = *k_leak_* ([Ca_ER_] – [Ca_cyt_]) 7.1

ER Ca^2+^ buffer (J_ERB_) [5] 8

*J_ERB_* = – [Ca_ER_]*B_0_k_f_* + *B_1_k_b_*  8.1

d(*B_0_*)/dt = – *B_0_*[Ca_ER_]*k_f_* + *B_1_k_b_* 8.2

d(B1)/dt = – *B_1_k_b_* + *B_0_*[Ca_ER_]*k_f_* 8.3

***IP_3_ fluxes***

IP_3_ 3-kinase [6] 9

*J_3K_* = *V_3K_* Hill([IP_3_]^4^, *k_3K_*) [IP_3_]/([IP_3_] + *k_3K2_*) 9.1

IP_3_ 5-phosphatase [7–9] 10

*J_5P_* = *V_5P_* ([IP_3_] – [IP_3_]_base_) 10.1

PLC_δ_  [10] 11

*J_Pδ_* = *V_Pδ_* Hill([IP_3_]^2^, *k_Pδ_*) / (1 + [IP_3_]/*k_Pδ2_*) 11.1

IP_3_ receptor (J_IP3R_) [11] 12

*J_IP3R_* = *V_max_* (*m_∞_n_∞_h*)^3^ ([Ca_ER_] – [Ca_cyt_]) 12.1

d(*h*)/dt = *α_h_* (1 – *h*) – β_h_*h* + ζ(t) 12.2

< ζ(t)> = 0 12.3

< ζ(t) ζ(t`) > = (*α_h_* (1 – *h*) + *β_h_h*) δ(t – t`) / *n* 12.4

*d_i_* = *b_i_* / *a_i_* 12.5

*n_∞_* = [IP_3_] / [IP_3_] + *d_1_* 12.6

*m_∞_* = [Ca_cyt_] / [Ca_cyt_] + *d_5_* 12.7

*α_h_* = *a_2_d_2_* ([IP_3_] + *d_1_*) / ([IP_3_] + *d_3_*) 12.8

*β_h_* = *a_2_*[Ca_cyt_] 12.9

mGluR [8] 13

*J_mGluR_* = *V_mGluR_* Hill([Glu]^2^, *k_mGluR_*), in the presence of glutamate 13.1

*J_mGluR_* = *V_mGluR_* Hill([DHPG]^2^, *k_mGluR_*), in the presence of DHPG 13.2

***Gliotransmitter release***

The below equations describe the two synaptotagmins (*Syt4* & *Syt7*) that control Ca^2+^-mediated gliotransmission. A schematic of their kinetic model is in S3 Figure.

Synaptotagmin 4 (J_Syt4_) [1,12] 14

*J_syt4_* = – *S_0_*2*k_f4_*[Ca_cyt_] + *S_1_k_b4_* – *S_1_k_f4_*[Ca_cyt_] + *S_2_*2*k_b4_b_4_* 14.1

d(*S_0_*)/dt = – *S_0_*2*k_f4_*[Ca_cyt_] + S_1_*k_b4_* 14.2

d(*S_1_*)/dt = – *S_1_k_f4_*[Ca_cyt_] + *S_2_*2*k_b4_b_4_* – *S_1_k_b4_* + *S_0_*2*k_f4_*[Ca_cyt_] 14.3

d(*S_2_*)/dt = – *S_2_*2*k_b4_b_4_* + *S_1_k_f4_*[Ca_cyt_] 14.4

Synaptotagmin 7 (J_Syt7_) [1,12] 15

*J_syt7_* = –*Y_0_*5*k_f7_*[Ca_cyt_] + *Y_1_k_b7_* – *Y_1_*4*k_f7_*[Ca_cyt_] + *Y_2_*2*k_b7_b_7_* – *Y*_2_3*k_f7_*[Ca_cyt_] +

*Y_3_*3*k_b7_b_7_^2^* – *Y_3_*2*k_f7_*[Ca_cyt_] + *Y_4_*4*k_b7_b_7_^3^* – *Y_4_k_f7_*[Ca_cyt_] + *Y_5_*5*k_b7_b_7_^4^* 15.1

d(*Y_0_*)/dt = –*Y_0_*5*k_f7_*[Ca_cyt_] + *Y_1_k_b7_* 15.2

d(*Y_1_*)/dt = –*Y_1_*4*k_f7_*[Ca_cyt_] + *Y_2_*2*k_b7_b_7_* – *Y_1_k_b7_* + *Y_0_5k_f7_*[Ca_cyt_] 15.3

d(*Y_2_*)/dt = –*Y_2_*3*k_f7_*[Ca_cyt_] + *Y_3_*3*k_f7_b_7_^2^* – *Y_2_*2*k_b7_b_7_* + (*Y_1_*4*k_f7_*[Ca_cyt_] 15.4

d(*Y_3_*)/dt = –*Y_3_*2*k_f7_*[Ca_cyt_] + *Y_4_*4*k_b7_b_7_^3^* – *Y_3_*3*k_b7_b_7_^2^* + *Y_2_*3*k_f7_*[Ca_cyt_] 15.5

d(*Y_4_*)/dt = –*Y_4_k_f7_*[Ca_cyt_] + *Y_5_*5*k_b7_b_7_^4^* – *Y_4_*4*k_b7_b_7_^3^* + *Y_3_*2*k_f7_*[Ca_cyt_] 15.6

d(*Y_5_*)/dt = –*Y_5_*5*k_b7_b_7_^4^* + *Y_4_k_f7_*[Ca_cyt_] 15.7

***Glutamate dynamics***

Glutamate dynamics at the perisynaptic region around the astrocytic compartment is described by the below equation.

[Glu](t) = *Glu_max_* δ(t – t_r_) + [Glu] exp(*k_Glu_*t), t_r_ = glutamate release time 16

**References**

1. Nadkarni S, Bartol TM, Sejnowski TJ, Levine H. Modelling vesicular release at hippocampal synapses. PLoS Comput Biol. 2010;6. doi:10.1371/journal.pcbi.1000983

2. Bartol TM, Keller DX, Kinney JP, Bajaj CL, Harris KM, Sejnowski TJ, et al. Computational reconstitution of spine calcium transients from individual proteins. Front Synaptic Neurosci. 2015;7: 1–24. doi:10.3389/fnsyn.2015.00017

3. Swaminathan D, Ullah G, Jung P. A simple sequential-binding model for calcium puffs. Chaos. 2009;19. doi:10.1063/1.3152227

4. De Young GW, Keizer J. A single-pool inositol 1,4,5-trisphosphate-receptor-based model for agonist-stimulated oscillations in Ca2+ concentration. Proc Natl Acad Sci. 1992;89: 9895–9899. doi:10.1073/pnas.89.20.9895

5. Higgins ER, Cannell MB, Sneyd J. A buffering SERCA pump in models of calcium dynamics. Biophys J. 2006;91: 151–163. doi:10.1529/biophysj.105.075747

6. De Pittà M, Goldberg M, Volman V, Berry H, Ben-Jacob E. Glutamate regulation of calcium and IP3 oscillating and pulsating dynamics in astrocytes. J Biol Phys. 2009. doi:10.1007/s10867-009-9155-y

7. Bennett MR, Farnell L, Gibson WG. A quantitative model of purinergic junctional transmission of calcium waves in astrocyte networks. Biophys J. 2005;89: 2235–2250. doi:10.1529/biophysj.105.062968

8. Ullah G, Jung P, Cornell-Bell AH. Anti-phase calcium oscillations in astrocytes via inositol (1, 4, 5)-trisphosphate regeneration. Cell Calcium. 2006;39: 197–208. doi:10.1016/j.ceca.2005.10.009

9. De Pittà M, Ben-Jacob E, Berry H. G Protein-Coupled Receptor-Mediated Calcium Signaling in Astrocytes. Computational Glioscience. 2019. pp. 115–150. doi:10.1007/978-3-030-00817-8_5

10. Stamatakis M, Mantzaris N V. Modeling of ATP-mediated signal transduction and wave propagation in astrocytic cellular networks. J Theor Biol. 2006;241: 649–668. doi:10.1016/j.jtbi.2006.01.002

11. Shuai J-W, Jung P. Stochastic properties of Ca(2+) release of inositol 1,4,5-trisphosphate receptor clusters. Biophys J. 2002;83: 87–97. doi:10.1016/S0006-3495(02)75151-5

12. Sun J, Pang ZP, Qin D, Fahim AT, Adachi R, Südhof TC. A dual-Ca2+-sensor model for neurotransmitter release in a central synapse. Nature. 2007;450: 676–682. doi:10.1038/nature06308
