# Supplementary material for: Amyloid pathology disrupts gliotransmitter release in astrocytes
Source: PLoS Comput Biol. 2022 Aug 1;18(8):e1010334. doi: 10.1371/journal.pcbi.1010334 (PMC9371304; doi:10.1371/journal.pcbi.1010334)
Supplement: S2 Appendix — (DOCX) [file pcbi.1010334.s002.docx]

**S2 Appendix.** Estimation of the number of astrocytic compartments in the experimental data

We systematically extracted data from diverse experimental studies to refine the model parameters [1–4]. Despite differences in experimental settings, temporal release histograms of gliotransmitter releases were remarkably similar (**S2A Figure**). From the reported values of the imaging area (~ 1115 μm^2^) and TIRF evanescent field (~ 100 nm) by these studies, we calculated an average imaging volume of roughly 111 μm^3^ per astrocyte. Together with previous measurements on total astrocytic volume (24,465 μm^3^) and the number of processes (100,000) in the CA1 layer of the rodent hippocampus, we estimated between 400-500 processes within the imaging volume [5]. Cumulative distributions of release histograms from these studies also indicated that a maximum of ~ 400 vesicles are released within this volume (**S2B Figure**). We, therefore, collected data from 400 independent simulation trials of a single astrocytic process to match the model results with experimental data.

**References**

1. Marchaland J, Cali C, Voglmaier SM, Li H, Regazzi R, Edwards RH, et al. Fast subplasma membrane Ca2+ transients control exo-endocytosis of synaptic-like microvesicles in astrocytes. J Neurosci. 2008;28: 9122–9132. doi:10.1523/JNEUROSCI.0040-08.2008

2. Santello M, Bezzi P, Volterra A. TNFα Controls Glutamatergic Gliotransmission in the Hippocampal Dentate Gyrus. Neuron. 2011;69: 988–1001. doi:10.1016/j.neuron.2011.02.003

3. Calì C, Marchaland J, Regazzi R, Bezzi P. SDF 1-alpha (CXCL12) triggers glutamate exocytosis from astrocytes on a millisecond time scale: Imaging analysis at the single-vesicle level with TIRF microscopy. J Neuroimmunol. 2008;198: 82–91. doi:10.1016/j.jneuroim.2008.04.015

4. Domercq M, Brambilla L, Pilati E, Marchaland J, Volterra A, Bezzi P. P2Y1 receptor-evoked glutamate exocytosis from astrocytes: Control by tumor necrosis factor-α and prostaglandins. J Biol Chem. 2006;281: 30684–30696. doi:10.1074/jbc.M606429200

5. Halassa MM, Fellin T, Takano H, Dong J-HJ, Haydon PG. Synaptic Islands Defined by the Territory of a Single Astrocyte. J Neurosci. 2007;27: 6473–6477. doi:10.1523/JNEUROSCI.1419-07.2007
