# Supplementary material for: Amyloid pathology disrupts gliotransmitter release in astrocytes
Source: PLoS Comput Biol. 2022 Aug 1;18(8):e1010334. doi: 10.1371/journal.pcbi.1010334 (PMC9371304; doi:10.1371/journal.pcbi.1010334)
Supplement: S3 Appendix — (DOCX) [file pcbi.1010334.s003.docx]

**S3 Appendix.** Calcium sensors in the gliotransmitter release model

Two synaptotagmins, *Syt4 and Syt7*, have been reported in astrocytes [1–3]. *Syt4* has a single low-affinity domain (C_2_B) that binds 2 Ca^2+^ ions with fast forward rates⁠. In contrast, *Syt7* has two calcium domains (C_2_A and C_2_B) that have high affinity but slow forward reaction rates [4]. This makes *Syt7* a slow calcium sensor that operates under low Ca^2+^ levels. TIRF imaging studies using fluorescently labeled vesicles also indicate fast kiss-and-run-like confined releases alongside slow-spreading full fusions events [5,6]. It is also established that *Syt4* with a single Ca^2+^ binding domain can only promote kiss-and-run exocytosis [7]. Conversely, capacitance measurements in astrocytes that primarily reflects full fusion exocytosis estimated a Hill’s coefficient of 5 that perfectly match with the Ca^2+^ binding sites in *Syt7* [8]*.* Based on these findings, we modeled two separate Ca^2+^ sensors, *Syt4* and *Syt7* with distinct affinities for Ca^2+^ binding and independently mediate fast (kiss-and-run release) and slow (full fusion) gliotransmitter release respectively (S3 Figure). *Syt4* has a low affinity for Ca^2+^ binding and a fast reaction rate. It is primarily activated by sharp and high amplitude Ca^2+^ events that have been observed near the ER tubules. On the other hand, *Syt7* has high-affinity for Ca^2+^ binding and a slow reaction rate, thereby, making it sensitive to slow and low amplitude Ca^2+^ rises. Analogous to vesicle recycling at the presynaptic terminal, we further assumed that every vesicle once released from the astrocytic process is endocytosed into the mobile vesicle pool and later some of them into the docked vesicle pool (Figures 2A and 2B). Separately, it has also been suggested that kiss-and-run and full fusion may arise from discrete vesicle pools [6,9]. Estimates on release parameters including release rate, vesicles per process and rates of endocytosis and vesicle recycling were obtained from published literature and are detailed in S1 Table.

Both fast kiss-and-run-like confined releases and slow-spreading full fusions events are seen in TIRF imaging studies of astrocytic processes that used fluorescent labeling of vesicles [5,6]. Additionally, the presence of synaptotagmins *Syt4* and *Syt7,* calcium-sensing molecules that trigger vesicle fusion, have been reported in astrocytes [1,2,10]. *Syt4* has a single low-affinity domain (C2B) that binds two Ca^2+^ ions with fast forward calcium-binding rates⁠. It is therefore primarily activated by sharp and high amplitude Ca^2+^ events. In contrast, *Syt7* has two calcium domains (C2A and C2B) that have high affinity but slow forward reaction rates [4]. These biophysical properties make *Syt7* a slow calcium sensor that can trigger vesicle fusion at low Ca^2+^ concentrations. *Syt4* with a single Ca^2+^ binding domain exclusively promotes kiss-and-run exocytosis [7], indicating kiss-and-run in the astrocytes is coordinated by *Syt4* . On the other hand, capacitance measurements of full fusion exocytosis in astrocytes are a quantitative match to the Ca^2+^ binding in *Syt7* with an estimated Hill's coefficient of five [11], suggesting that *Syt7* governs full-fusion in astrocytes.

**References**

1. Mittelsteadt T, Seifert G, Alvárez-Barón E, Steinhäuser C, Becker AJ, Schoch S. Differential mRNA expression patterns of the synaptotagmin gene family in the rodent brain. J Comp Neurol. 2009;512: 514–528. doi:10.1002/cne.21908

2. Zhang Q, Fukuda M, Van Bockstaele E, Pascual O, Haydon PG. Synaptotagmin IV regulates glial glutamate release. Proc Natl Acad Sci. 2004;101: 9441–9446. doi:10.1073/pnas.0401960101

3. Rao SK, Huynh C, Proux-Gillardeaux V, Galli T, Andrews NW. Identification of SNAREs Involved in Synaptotagmin VII-regulated Lysosomal Exocytosis. J Biol Chem. 2004;279: 20471–20479. doi:10.1074/jbc.M400798200

4. Hui E, Bai J, Wang P, Sugimori M, Llinas RR, Chapman ER. Three distinct kinetic groupings of the synaptotagmin family: candidate sensors for rapid and delayed exocytosis. Proc Natl Acad Sci U S A. 2005;102: 5210–4. doi:10.1073/pnas.0500941102

5. Malarkey EB, Parpura V. Temporal characteristics of vesicular fusion in astrocytes: Examination of synaptobrevin 2-laden vesicles at single vesicle resolution. J Physiol. 2011;589: 4271–4300. doi:10.1113/jphysiol.2011.210435

6. Bowser DN, Khakh BS. Two forms of single-vesicle astrocyte exocytosis imaged with total internal reflection fluorescence microscopy. Proc Natl Acad Sci. 2007;104: 4212–4217. doi:10.1073/pnas.0607625104

7. Wang CT, Lu JC, Bai J, Chang PY, Martin TFJ, Chapman ER, et al. Different domains of synaptotagmin control the choice between kiss-and-run and full fusion. Nature. 2003;424: 943–947. doi:10.1038/nature01857

8. Kreft M, Stenovec M, Rupnik M, Grilc S, Kržan M, Potokar M, et al. Properties of Ca2+-dependent exocytosis in cultured astrocytes. Glia. 2004;46: 437–445. doi:10.1002/glia.20018

9. Marchaland J, Cali C, Voglmaier SM, Li H, Regazzi R, Edwards RH, et al. Fast subplasma membrane Ca2+ transients control exo-endocytosis of synaptic-like microvesicles in astrocytes. J Neurosci. 2008;28: 9122–9132. doi:10.1523/JNEUROSCI.0040-08.2008

10. Martinez I, Chakrabarti S, Hellevik T, Morehead J, Fowler K, Andrews NW. Synaptotagmin vii regulates Ca2+-dependent exocytosis of lysosomes in fibroblasts. J Cell Biol. 2000;148: 1141–1149. doi:10.1083/jcb.148.6.1141

11. Zhang Q, Pangršič T, Kreft M, Kržan M, Li N, Sul JY, et al. Fusion-related Release of Glutamate from Astrocytes. J Biol Chem. 2004;279: 12724–12733. doi:10.1074/jbc.M312845200
