# Supplementary material for: Amyloid pathology disrupts gliotransmitter release in astrocytes
Source: PLoS Comput Biol. 2022 Aug 1;18(8):e1010334. doi: 10.1371/journal.pcbi.1010334 (PMC9371304; doi:10.1371/journal.pcbi.1010334)
Supplement: S1 Fig — (DOCX) [file pcbi.1010334.s004.docx]

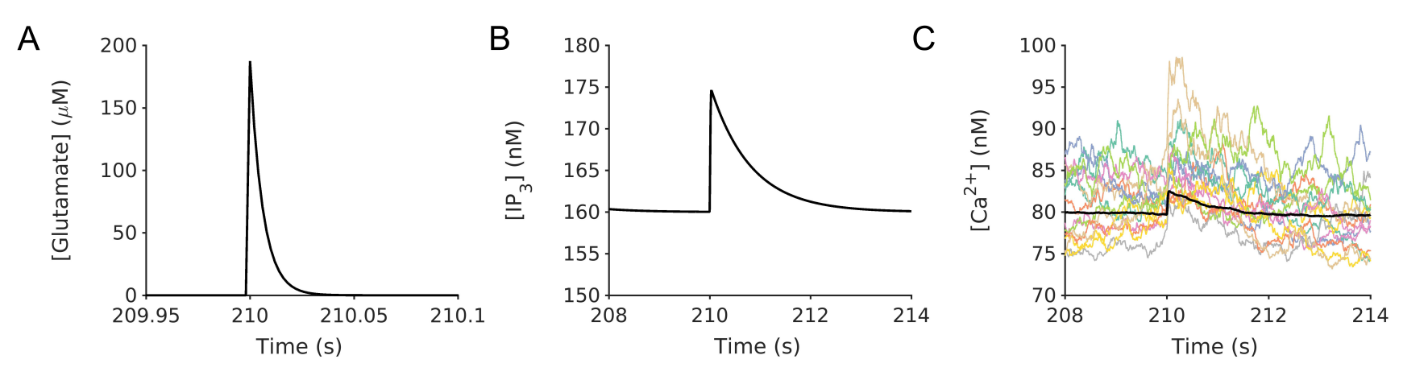


**S1 Figure.** Time courses of glutamate, IP_3_ and Ca^2+^ concentrations in a single astrocytic microdomain. (A) Temporal profile of glutamate concentration that captures the experimentally observed peak, risetime and decay of perisynaptic glutamate levels following the release of a single vesicle. (B) The time course of IP_3_ in response to mGluRs activation by a single glutamate vesicle. (C) Representative stochastic Ca^2+^ events evoked by the glutamate concentration profile as shown in A.
