# Supplementary material for: Amyloid pathology disrupts gliotransmitter release in astrocytes
Source: PLoS Comput Biol. 2022 Aug 1;18(8):e1010334. doi: 10.1371/journal.pcbi.1010334 (PMC9371304; doi:10.1371/journal.pcbi.1010334)
Supplement: S2 Fig — (DOCX) [file pcbi.1010334.s005.docx]

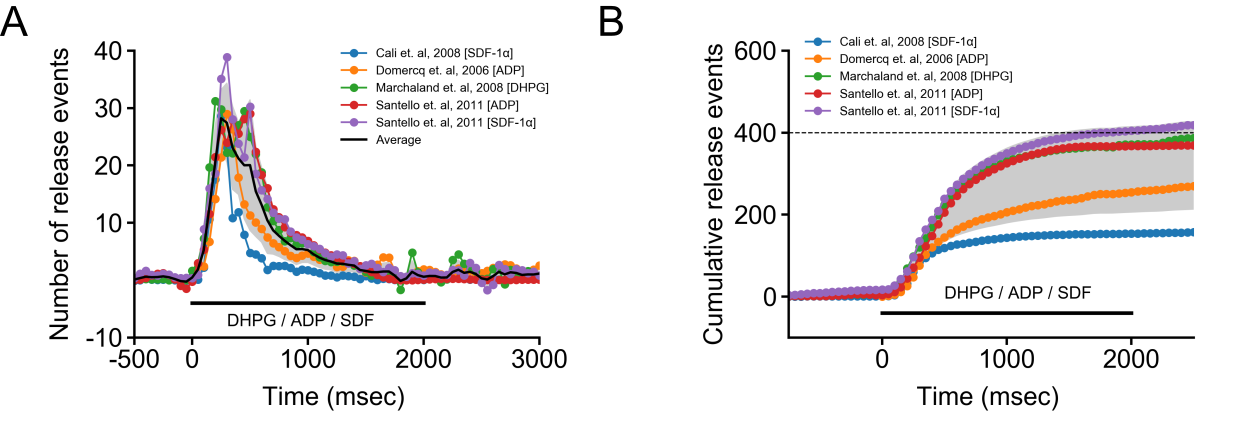


**S2 Figure**. Temporal profiles of vesicle release from astrocytic domains in response to various stimuli. (A) Release histograms from astrocytic sub-domains stimulated with either DHPG (an mGluR1/5 agonist), SDF-1α (CXCR4 receptor agonist) or 2MeSADP (a P2Y1R agonist) exhibit a remarkable similarity in their time courses. (B) Cumulative distribution of vesicle release from astrocytes when stimulated with different agonists. All data were captured directly from figures in published studies [1–4].

**References**

1. Calì C, Marchaland J, Regazzi R, Bezzi P. SDF 1-alpha (CXCL12) triggers glutamate exocytosis from astrocytes on a millisecond time scale: Imaging analysis at the single-vesicle level with TIRF microscopy. J Neuroimmunol. 2008;198: 82–91. doi:10.1016/j.jneuroim.2008.04.015

2. Domercq M, Brambilla L, Pilati E, Marchaland J, Volterra A, Bezzi P. P2Y1 receptor-evoked glutamate exocytosis from astrocytes: Control by tumor necrosis factor-α and prostaglandins. J Biol Chem. 2006;281: 30684–30696. doi:10.1074/jbc.M606429200

3. Marchaland J, Cali C, Voglmaier SM, Li H, Regazzi R, Edwards RH, et al. Fast subplasma membrane Ca2+ transients control exo-endocytosis of synaptic-like microvesicles in astrocytes. J Neurosci. 2008;28: 9122–9132. doi:10.1523/JNEUROSCI.0040-08.2008

4. Santello M, Bezzi P, Volterra A. TNFα Controls Glutamatergic Gliotransmission in the Hippocampal Dentate Gyrus. Neuron. 2011;69: 988–1001. doi:10.1016/j.neuron.2011.02.003
