# Supplementary material for: Amyloid pathology disrupts gliotransmitter release in astrocytes
Source: PLoS Comput Biol. 2022 Aug 1;18(8):e1010334. doi: 10.1371/journal.pcbi.1010334 (PMC9371304; doi:10.1371/journal.pcbi.1010334)
Supplement: S3 Fig — (DOCX) [file pcbi.1010334.s006.docx]

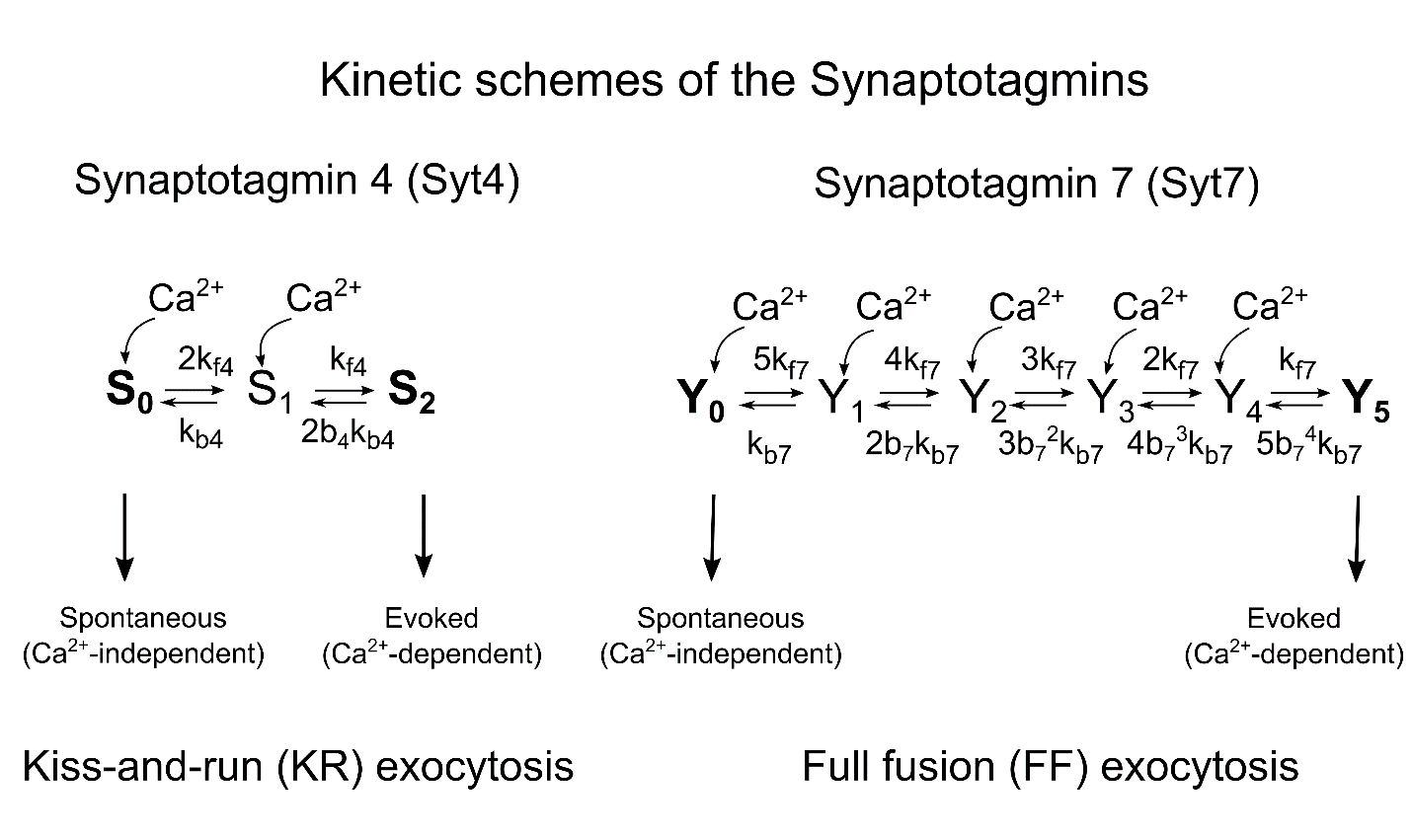


**S3 Figure**. The gliotransmission model has two synaptotagmins (*Syt4* & *Syt7*). *Syt4* has two Ca^2+^ binding sites whose affinity for Ca^2+^ is low compared to *Syt7*, while *Syt7* has 5 Ca^2+^ binding domains. S_0_, Y_0_: states that cause spontaneous, Ca^2+^-independent release at low rates. S_2_, Y_5_: states that cause Ca^2+^-dependent release. *Syt4* releases docked vesicles via kiss-and-run mode, while *Syt7* releases mobile vesicles via the full fusion pathway. A detailed description of the synaptotagmins is in S3 Appendix and the parameters are in S1 Table.
