# Supplementary material for: Amyloid pathology disrupts gliotransmitter release in astrocytes
Source: PLoS Comput Biol. 2022 Aug 1;18(8):e1010334. doi: 10.1371/journal.pcbi.1010334 (PMC9371304; doi:10.1371/journal.pcbi.1010334)
Supplement: S1 Table — (DOCX) [file pcbi.1010334.s007.docx]

**S1 Table.** Model parameters

| **No.** | **Component name** | **Parameters** |
| --- | --- | --- |
| **Calcium dynamics** | | |
| 4 | PMCA [1] | *k_f1_* = 15 µM^-1^s^-1^, *k_b_ =* 20 s^-1^, *k_f2_* = 20 s^-1^, *k_f3_ =* 100 s^-1^*, k_l_* = 0.6 s^-1^, *PMCA density* = 400 µm^-2^  For Aβ conditions: *k_b_* = 60 s^-1^ |
| 5 | Cytosol Ca^2+^ buffer [2] | *k_f_ =* 60 µM^-1^s^-1^*, k_b_ =* 1200 s^-1^*,*  Total buffer concentration *=* 50 µM |
| 6 | SERCA [3,4] | *V_SERCA_ =* 250 µMs^-1^, *k_a_* = 100 nM |
| 7 | ER-leak [5] | *k_leak_ =* 0.2 s^-1^ |
| 8 | ER Ca^2+^ buffer [6] | *k_f_ =* 1 µM^-1^s^-1^*, k_b_ =* 80 s^-1^*,*  *buffer concentration =* 10 µM |
| **IP_3_ dynamics** | | |
| 9 | IP_3_ 3-kinase [7] | *V_3K_ =* 5 µMs^-1^, *k_3K_* = 0.4 µM, *k_3K2_* = 10 µM |
| 10 | IP_3_ 5-phosphatase [3,8] | *V_5P_ = 1*.25 s^-1^ |
| 11 | PLC_δ_ [8] | *V_Pδ_ =* 0.02 µMs^-1^, *k_Pδ_* = 1 µM, *k_Pδ2_* = 1.5 µM |
| **Receptors** | | |
| 12 | IP_3_ receptor [9,10] | *a_1_* = 400 µMs^-1^, *a_2_* = 0.2 µM^-1^s^-1^, *a_3_* = 400 µM^-1^s^-1^, *a_4_* = 0.2 µM^-1^s^-1^, *a_5_* = 20 µM^-1^s^-1^, *b_1_* = 52 s^-1^, *b_2_* = 0.2 s^-1^, *b_3_* = 377.36 s^-1^, *b_4_* = 0.02 s^-1^, *b_5_* = 1.64 s^-1^, *V_max_* = 6.0 s^-1^, *n* = 5 |
| 13 | mGluR [3] | For normal condition:  *V_mGluR_ =* 0.65 µMs^-1^,  *k_mGluR_* = 6 µM (DHPG), 11 µM (Glutamate)  For Aβ conditions:  *V_mgluR_* = 1.4µMs^-1^, *k_mGluR_* = 3 µM |
| **Gliotransmitter release machinery** | | |
| 14 | Syt4 [11] | *k_f4_ =* 153 µM^-1^s^-1^, *k_b4_ =* 3500 s^-1^, *b_4_ =* 0.25,  *r_04_ =* 0.000417 s^-1^, *r_ca4_ =* 115 s^-1^, |
| 15 | Syt7 [11] | *k_f7_ =* 3.82 µM^-1^s^-1^, *k_b7_ =* 60 s^-1^, *b_7_ =* 0.25,  *r_07_ =* 0.000417 s^-1^, *r_ca7_ =* 8 s^-1^ |
| 16 | Glutamate dynamics | *Glu_max_* = 200 µM, *k_Glu_* = 160 s^-1^ |
| 17 | Fraction of docked vesicles [12] | *N_doc_* = 0.8 |
| 18 | Fraction of mobile vesicles (*N_mob_*) [12] | 0.2 |
| 19 | Kiss-and-run release endocytosis rate (*k_e4_*) [13] | 0.66 s^-1^ |
| 20 | Kiss-and-run release reacidification rate (*k_a4_*) [13] | 0.6 s^-1^ |
| 21 | Full fusion release endocytosis rate (*k_e7_*) [13] | 0.16 s^-1^ |
| 22 | Full fusion release reacidification rate (*k_a7_*) [13] | 0.052 s^-1^ |
| 23 | Mobile vesicle replenishment rate (k*_mob_*) [13] | 0.615 s^-1^ |
| 24 | Vesicle docking rate (*k_doc_*) [13] | 0.75 s^-1^ |
| **Additional model parameters** | | |
| 25 | Resting cytosol Ca^2+^ [14] | 80 nM |
| 26 | Resting ER Ca^2+^ [4] | 400 µM |
| 27 | Resting IP_3_ ([IP_3_]_base_) [15] | 160 nM |
| 28 | External Ca^2+^ [16] | 2 mM |
| 29 | Cytosol volume [17] | 0.37 µm^3^ |
| 30 | Cytosol area to volume ratio [17,18] | 13.3 µm^-1^ |
| 31 | ER to cytosol volume ratio [13] | 0.5 |
| 32 | ER to cytosol area ratio [17,18] | 10 |
| 33 | Specific capacitance [18] | 1.0 µF cm^-2^ |

**References**

1. Nadkarni S, Bartol TM, Sejnowski TJ, Levine H. Modelling vesicular release at hippocampal synapses. PLoS Comput Biol. 2010;6. doi:10.1371/journal.pcbi.1000983

2. Bartol TM, Keller DX, Kinney JP, Bajaj CL, Harris KM, Sejnowski TJ, et al. Computational reconstitution of spine calcium transients from individual proteins. Front Synaptic Neurosci. 2015;7: 1–24. doi:10.3389/fnsyn.2015.00017

3. Swaminathan D, Ullah G, Jung P. A simple sequential-binding model for calcium puffs. Chaos. 2009;19. doi:10.1063/1.3152227

4. Burdakov D, Petersen OH, Verkhratsky A. Intraluminal calcium as a primary regulator of endoplasmic reticulum function. Cell Calcium. 2005;38: 303–310. doi:10.1016/j.ceca.2005.06.010

5. De Young GW, Keizer J. A single-pool inositol 1,4,5-trisphosphate-receptor-based model for agonist-stimulated oscillations in Ca2+ concentration. Proc Natl Acad Sci. 1992;89: 9895–9899. doi:10.1073/pnas.89.20.9895

6. Higgins ER, Cannell MB, Sneyd J. A buffering SERCA pump in models of calcium dynamics. Biophys J. 2006;91: 151–163. doi:10.1529/biophysj.105.075747

7. De Pittà M, Goldberg M, Volman V, Berry H, Ben-Jacob E. Glutamate regulation of calcium and IP3 oscillating and pulsating dynamics in astrocytes. J Biol Phys. 2009. doi:10.1007/s10867-009-9155-y

8. Stamatakis M, Mantzaris N V. Modeling of ATP-mediated signal transduction and wave propagation in astrocytic cellular networks. J Theor Biol. 2006;241: 649–668. doi:10.1016/j.jtbi.2006.01.002

9. Shuai J-W, Jung P. Stochastic properties of Ca(2+) release of inositol 1,4,5-trisphosphate receptor clusters. Biophys J. 2002;83: 87–97. doi:10.1016/S0006-3495(02)75151-5

10. Holtzclaw LA, Pandhit S, Bare DJ, Mignery GA, Russell JT. Astrocytes in adult rat brain express type 2 inositol 1,4,5-trisphosphate receptors. Glia. 2002;39: 69–84. doi:10.1002/glia.10085

11. Sun J, Pang ZP, Qin D, Fahim AT, Adachi R, Südhof TC. A dual-Ca2+-sensor model for neurotransmitter release in a central synapse. Nature. 2007;450: 676–682. doi:10.1038/nature06308

12. Kreft M, Stenovec M, Rupnik M, Grilc S, Kržan M, Potokar M, et al. Properties of Ca2+-dependent exocytosis in cultured astrocytes. Glia. 2004;46: 437–445. doi:10.1002/glia.20018

13. Marchaland J, Cali C, Voglmaier SM, Li H, Regazzi R, Edwards RH, et al. Fast subplasma membrane Ca2+ transients control exo-endocytosis of synaptic-like microvesicles in astrocytes. J Neurosci. 2008;28: 9122–9132. doi:10.1523/JNEUROSCI.0040-08.2008

14. Kuchibhotla K V., Lattarulo CR, Hyman BT, Bacskai BJ. Synchronous Hyperactivity and Intercellular Calcium Waves in Astrocytes in Alzheimer Mice. Science (80- ). 2009;323: 1211–1215. doi:10.1126/science.1169096

15. Wade JJ, McDaid LJ, Harkin J, Crunelli V, Kelso JAS. Bidirectional coupling between astrocytes and neurons mediates learning and dynamic coordination in the brain: A multiple modeling approach. PLoS One. 2011;6: 1–24. doi:10.1371/journal.pone.0029445

16. Henckens MJAG, van der Marel K, van der Toorn A, Pillai AG, Fernández G, Dijkhuizen RM, et al. Stress-induced alterations in large-scale functional networks of the rodent brain. Neuroimage. 2015;105: 312–322. doi:10.1016/j.neuroimage.2014.10.037

17. Grosche A, Grosche J, Tackenberg M, Scheller D, Gerstner G, Gumprecht A, et al. Versatile and Simple Approach to Determine Astrocyte Territories in Mouse Neocortex and Hippocampus. PLoS One. 2013;8. doi:10.1371/journal.pone.0069143

18. Østby I, Øyehaug L, Einevoll GT, Nagelhus EA, Plahte E, Zeuthen T, et al. Astrocytic mechanisms explaining neural-activity-induced shrinkage of extraneuronal space. PLoS Comput Biol. 2009;5. doi:10.1371/journal.pcbi.1000272
