# Supplementary material for: Amyloid pathology disrupts gliotransmitter release in astrocytes
Source: PLoS Comput Biol. 2022 Aug 1;18(8):e1010334. doi: 10.1371/journal.pcbi.1010334 (PMC9371304; doi:10.1371/journal.pcbi.1010334)
Supplement: Attachment — Submitted filename: Pillai.PCOMPBIOL-D-20-01194.pdf [file pcbi.1010334.s008.pdf]

PCOMPBIOL-D-20-01194
Title: Amyloid pathology disrupts gliotransmitter release in astrocytes
Authors: A. G. Pillai and S. Nadkarni

Overview
Pillai and Nadkarni introduce a novel model of astrocyte-synapse interactions that revolves around the biophysical description of calcium-dependent glutamate release from astrocytes. In particular, they distinguish between different mechanisms of glutamate exocytosis – kiss-and-run vs. full-fusion – by considering the Ca2+-dependent dynamics of different synaptotagmins. Then, they proceed to mimic amyloid-like pathology changing values to model parameters, and documenting, through numerical simulations, how amyloid-beta could affect astrocytic physiology, and astrocytic glutamate release in particular. The study is original and of interest to PLoS Computational Biology, with the potential to reach out to a broad audience, comprising both experimentalists and computationalists. Nonetheless, there is ample space for improvement of the manuscript in its present version. There are at least three main issues with the present paper. First, the research question is not clear, and the way the material is exposed is not exhaustive and somehow lousy. Second, there is a critical issue with the references cited by the authors, which often do not support the authors' claims. Third, the writing style is poor, making the reading of the manuscript hard for several broken sentences, ambiguous terminology, and logical flow that is not consistent. For these reasons, I am advising for major revisions, which I am following detailing.

Suggestions for editing.
Four main aspects should be first revised:

1. The research question and aim of the study should be clearly stated.
2. References and citations should be adjusted accordingly to the claims they associated with. This should go along with a rephrasing of Introductions and strengthening of Discussions.
3. Presentation of the model equations should be detailed in an appendix at the end of the paper, rather than in tables.
4. Figures should be overall improved.

Because you have not provided a reviewer-friendly manuscript with line numbers, I will limit my remarks to elaborating on these four points only. In doing so I am leaving out many grammar aspects – suggesting the use of online tools (e.g., Grammarly) or professional English services offered by the journal. I hope that on your next submission, you will mind about these aspects as well. Put simply, the manuscript is written in poor English, with many excruciating grammar issues.

Research question and aim of the study.
You fail to state your research question accurately. In the Introduction, you emphasize astrocytic calcium and how such signaling pathway could be affected by amyloid-related pathology. But your main results show how disruption of such signaling could dramatically change the modes of glutamate exocytosis from an astrocyte. You are missing to explain to the reader in the Introduction why we should care about gliotransmitter release from the astrocyte in the context of Alzheimer's. In doing so, there is a mismatch between Introduction and discussion, which should be resolved accordingly.

References.

I am proceeding progressively, mentioning references as they appear in your manuscript.

- Refs. 1–4 are not proper. You need reviews here. And, as much as possible, from the past decade, that is, not older than 2010, please.
- Ref. 5 is not about gliotransmitter release but discusses the sources of variability of calcium in astrocytes. Also, why are you limiting your reasoning to the hippocampus at this stage?
- Ref. 10 is a bit old an should be complemented by Bazarghani and Attwell, Nature Neurosci. 2016. Similarly, when you say "fast, high-amplitude and highly compartmentalized Ca2+ signals," references 11 and 12 are not correct. Those works did not focus on calcium signals. Moreover, when talking about calcium compartmentalization, those references are still not appropriate. Again, use Bazarghani and Attwell and references within to properly support your claim.
- Since most of your modeling work relies on Ref. 12, it is probably worth explaining it more. However, linking calcium machinery and the existence of different exocytotic mechanisms by mGluRs and ER clustering is not by Refs. 12 and 13. Instead, the way to do it is to look into the last part of Ref. 5 and take into account the recent work by Volterra and Savtchouk in J. Neurosci 2018.
- In the presentation of Synaptotagmins, why should we care about Syt1? I assume because this is the main one involved in synaptic neurotransmitter release, but then you should state it and provide essential references. Ref. 17 is in cerebellar astrocytes: not what you want if you are considering the hippocampus.
- "Equipped with this elaborate machinery for exocytosis that is analogous to the presynaptic terminal in neurons" – a reference is missing here.
- There is ambiguity in your exposition of astrocyte-mediated synchrony. Ref. 19 deals with SICs and looks at spontaneous calcium events rather than evoked ones; hence it is not appropriate. Also, it is not clear whether SICs, and neuronal synchrony thereby, is by exocytosis of glutamate by astrocytes. Finally, in your results, you are looking at the synchronization of glutamate release with *upstream* neural activity, and not, as the studies that you refer to point to, to synchronization of downstream neuronal firing with respect to calcium-dependent SICs/glutamate release. Be careful: the field is evolving rapidly, and these details are crucial nowadays with respect to 10 years ago.
- The sentence associated with Refs. 21,22 can be dropped.
- Ref. 28 is not related to any aspect of pathology. You should look instead into work by Molofsky or Khakh for more appropriate work.
- Ref 29 is also not appropriate. You should refer here to the original studies that identified Orai1 and STIM1 channels.
- Ref. 31: why should we consider a study in neurons, if you are reasoning on astrocytes?
- Refs. 26,34 following "As a result, ER resting Ca2+ levels…" are not representative of previous models: the literature on calcium signaling is vast, and either you refer to relevant reviews here, or you look into studies that you directly use/extend in your modeling work.
- When discussing Ref. 34, note that you are not using a "Langevin approximation" – there is no such thing. Instead, you are using a Langevin description for stochastic IP3R gating.

- When discussing IP3 dynamics, following the description of PLC-delta, IP3-3K, and IP-5P modeling require the citation of Matrosov et al., Computational Glioscience 2019.
- Ref. 36 should also be complemented by the more recent work by those investigators in Computational Glioscience 2019. This latter reference and the citations within will likely tell you why a statement such as “Ca2+-independent IP-5P-mediated degradation” is not correct.
- Ref. 12 about vesicle distribution and kinetics in the first paragraph of the section “Gliotransmitter Release” is not appropriate. Arguably, works by Zorec and Parpura should be cited instead. The same Ref. is inappropriate at the end of the first sentence on page 9.
- Ref. 44 on page 11 is not sufficient. You should refer to De Pitta in Computational Glioscience 2019 to accurately estimate the concentration range of Ca2+ threshold for gliotransmitter exocytosis.
- Refs. 50 and 51 (p15) – I am missing the logical link: I do not see how these two references are related to your current work. The whole sentence they refer to should be moved to Methods.
- Ref 43 is not appropriate for the temporal profile of extracellular glutamate. For example, you should refer to other studies by Clements et al., (1992-1996); Rusakov and co. (2000-2004) or, more recently, De Pitta in Computational Glioscience 2019).
- Ref.28 on p21 is not appropriate. A Review by Perea and co-workers could instead be more reasonable. Or work by Savtchouk and Volterra, JN 2018; De Pitta et al., 2015; Araque et al., 2014. The sense of the sentence associated with this reference is also wrong. Astrocytes modulate synaptic transmission, and this can result in neuronal synchrony, and not the opposite. If you are looking at independent mechanisms, then gliotransmission is not the common pathway. You will need to look into ion homeostasis (see for example, work by Nedergaard’s group).
- Ref. 63 is Ref 12.
- Refs. 19,65 on p24 are not appropriate. Angulo’s work is not on calcium waves, and synchronization is not the main result. Lavrentovich’s study instead is a straightforward one on calcium waves, but not in a biophysical way as yours. Also, your model does not account for calcium waves.
- Refs. 12,66 on the second row on p25 are not appropriate. Microdomains in vitro are not relevant. Marchaland’s work was not on microdomains. Covelho’s work was on different types of gliotransmitter release from the same astrocyte. Di Castro’s work is appropriate but should be cited along with Panatier’s companion paper in Neuron 2011.
- Ref. 4 on p25 for the structural information on the mobile vesicle pool is not appropriate. Jourdain’s work was not dealing with any characterization of such kind, and they never proved the existence of a vesicle pool – this is, in fact, an open question in the field, and an ansatz in your modeling work.

Model Presentation.

- At the end of the paper, I want to read an appendix that reflects the different subsections of “Methods/Models,” and explains individual model’s modules. Then, you append one single long table, with different modules separated by sections, for all the parameters, and your choice of values, but not equations. Finally, a second appendix, or each of the subsections of the former appendix, should clarify your choice of all model’s parameters / or parameters related to that

specific module, providing the necessary references. With this regard, note that the long intermediate paragraph on page 9, for example, is more appropriate within a section that explains how you estimate parameters in your model.

- Although you refer to your model as a model of the tripartite synapse, in practice, your model is a model of an astrocytic compartment, and glutamate release from there. A further source of ambiguity is that you consider only a single astrocytic compartment, but then, quite often in your simulations, you refer to "astrocytic processes" in a misleading fashion. You do not model many different processes: the source of variability in your simulations comes from the stochastic opening of IP3Rs. Please amend your text accordingly, and try to be as precise as possible, avoiding ambiguities.
- Be careful in your equation 3: there is no such thing as IP3K or IP3P: the correct abbreviations are IP3-3K and IP-5P.
- You introduce an IP3-base in your equation for IP-5P degradation, but are you sure that in your simulations IP3>= IP3-base? If this is not the case, your J_5P will become a production term. Please clarify, and amend your equations accordingly if needed.
- On page 9, you write three times "data not shown" on crucial aspects of your model setup. Please, provide these data instead as supplementary figures. This will help to clarify your procedure, ensuring reproducibility.
- In the bottom paragraph on page 9, what is "tau"? I do not see any equation that includes it.
- You are making some confusion in the notation. Hill functions, as well as EC50, are used exclusively in chemical/binding reactions. Other formulas that are identical to Hill functions, but are not in terms of chemical equations, are only rational sigmoid functions. This is the case of eq. 5, for example.
- Similarly, there is not such a thing as EC50 in Hz. EC50, as the acronym says, stands for half-maximal "effective concentration." Please amend your notation in formulas and text accordingly.
- You tend to overuse parentheses. You can easily simplify your notation, dropping parentheses from Eqs. 2, 3, 8, 9. Also, be consistent with your notation, please: if Ca2+ denotes a concentration, then always use [Ca2+], and append as a subscript out of the right bracket the relevant identifier, i.e. [Ca2+]_cyt. Amend accordingly, Eqs. 1,2,4,6.
- I do not understand your eq. 6. Can you please use standard notation? What is "÷"? If it is division, use the horizontal bar, and write your formulas by \frac{}{} in the standard form.
- Eq. 8. Use \frac{1}{N_{j,m}}. What about the index of the sum?
- The Section "Computational of correlation between Ca2+ and gliotransmitter release events" could be dropped (or lumped in a Supplementary text). See my comments on "Results and Figures." If you decide to retain it, explain the several passages by formulas rather than verbally, which will ease its accessibility. Eq. 9 has several typos on the \cdot.
- Numerical simulations: You should also lump here Data availability. How is the code implemented? There is no need to reference "Python" but you need to say what version you are using. Instead, you need to cite MATLAB properly as documented on the web, specify the release version, and note that MATLAB is a ®.
- The overall description of docked/fixed, transporter/mobile vesicles' pools is confusing, since you often exchange the descriptors.

Results and Figures.

- You limit your analysis to a mere description of your simulations' results, failing to give essential insights on the biophysical mechanisms that, in the model, account for your predictions – and how these predictions reflect in the real brain / AD pathology. For example, on page 17: when you say that you have increased resting Ca2+ levels but not IP3 levels (Figures 3C – E), so what? Why should we care? Another example on p 18: "Both frequency and amplitude of Ca2+ events were enhanced in the AD ~~astrocytic process~~ [note the misuse of "astrocytic process"] compared to the control group. Additionally, we also observed spontaneous Ca2+ events in AD groups with impaired PMCAs" – so what? On p19, "Notably impaired PMCA activity, apart from increasing spontaneous activity (repetition!), also lowered rise time… – so what? What does this imply in terms of pathology? Why should we care? How do you link these results with experiments? There are many further examples in your results that ask for these questions and remained unanswered. Another important example always on p19: "However, unlike in the AD-mGluR groups, impairment in PMCA functionality was associated with a substantial flattening of kiss-and-run release rates" – ok, but why? What is happening in your model? What are the biophysical underpinnings? p21 "Our results ~~from the model~~ indicate that despite… "so what?
- Talking about synchrony on p20. Aside from the ambiguity of how you look at coherence – coherence of what w.r.t. what? – the critical point here is that the only source of variability is the stochastic equation for h in the IP3R model. What happens if you drop it? Can you please repeat your simulations and analysis of synchrony/coherence without noise first?
- You tend to confuse Amyloid-beta pathology with AD. The two things are different: AD is an amyloid-beta pathology, but not all amyloid-beta pathologies are AD. Please avoid ambiguity and clarify.
- You describe the increase of resting Ca2+ as a side effect, but it is not. This is a crucial aspect that explains your results related to the alteration of PMCA in terms of calcium signaling and downstream gliotransmitter release in amyloid-related pathology. This also explains why your results in 4K are not surprising.
- You often say that you apply DHPG to your model. But there is no explanation of how this is achieved in your simulations. You only mention glutamate. I assume that you are just applying a step stimulus, and you imagine that it is equivalent to DHPG in Marchaland et al.' s experiments. If so, please state it, and avoid ambiguities. Also, you should bear in mind that mGluR affinity changes between glutamate and DHPG.
- There is a problem with your figures. First, I do not understand whether it is a platform issue, or you have uploaded the figures as I have them effectively, but their resolution is low. They seem to have been uploaded as PNG. This is not the ideal format: you should provide them in PDF or EPS. If you are going for PNG, then provide them with a resolution of at least 1200x1200. The ones that I have are barely readable.
- Have you contacted the authors to re-use their data from Ref. 12? How did you obtain those data? I do not see any credits in the manuscript.
- p23: "To examine this link deeper… and release events in AD astrocytes is a direct outcome of the rapid depletion of docked vesicles" – not clear where and how you see it.

- Figure 1: Instead of using bar plots for your simulated data, use points. Also, plot error bars accordingly. This will apply for panels D–F. The legend should appear in the first plot (D). You may re-arrange in a matrix of 2x3 instead of 3x2.
- Figure 2: The kinetic schemes should be included in the appendix when you present the model equations for the Syts. A and B panels should be complemented by sample simulations for the two Syts' kinetics to clarify how they work in the model. Be consistent with your notation: In the text, you use SytX, but in the figure you use Syt-X – perhaps move 3G-H here?
- Figure 3: Panel B: Slope is misleading: I assume you mean "coefficient." Panels B – C: where are the error bars on your data points? What do data points vs. dashed lines represent? Panel D, provide axes, please, and plot the raster image in full size as the other panels. Avoid Blue v. Red as they are rendered identically in grayscale. Panels E and F: same considerations as for 1 D–F.
- Figure 4: Panels A, B, and J, K: What do data points vs. dashed lines represent? Panels A, B: where are the error bars? Panel B: Why two sets of vertical lines? And why negative PMCA current? You can simplify the y-axis scale, using uM/s units instead, and reporting the absolute PMCA-mediated flux. Panels C – E: where are the error bars? Please, specify bar labels in the x-axis for all plot, instead of having labels inside the bars. You can also consider lumping the three histograms into a single bar-plot differentiating among the three variables. Panels F – I: are you using the same stimulus/seed for the random generator for the same colors across different panels? Otherwise, the results are not technically, same colors, but completely different simulations.
- Figure 5: Panels A – D: same considerations of 4F – I. Also, why are you considering so many traces here and not as many as in the previous figures? Please try to be consistent. Panels E and F: where are the error bars? Panels G and H: How are you computing the FWHM and the decay time constant? There is no explanation in your methods.
- Figure 6: Use a 2x 4/3 matrix instead of a 4x 2/1 configuration. Panels A – D: please provide them with axes. I do not understand what the gray bar on the bottom is.
- Figure 7: Polar plots in Panels A and C are hardly readable and informative. I would advise dropping them or provide them as full histograms from 0 to 360 degrees. I would keep only Panels B and C, and lump them along with Panels 8E – G in a new Figure 7. Alternatively, since there is no reason why you want to show only the phase difference for 0.2 Hz rather than for the whole frequency spectrum, you may provide heatmaps in terms of phi vs. nu. Panels B and D: where are the error bars? No need to repeat the legend in these two plots.
- Figure 8: Panels A – D can be dropped: they are hardly informative. Also, your scale choice for the color bar makes your results scarcely interesting. You may try with a log scale. I would drop this analysis. Panels E – G: where are the error bars?

Discussion.

- Your discussion is lacking structure, possibly because you failed initially to clarify your research question. Moreover, you fail to put your results in perspective, mostly restating the result rather than elaborating on the functional/pathological implications. A typical example is on p24 when you state that "one of the most important insights (actually predictions) of this study is the observed loss of temporal precision of individual Ca2+ (events) and vesicle release in AD-like astrocytes" Ok, but why is this important for the pathology? Please elaborate.

- On p25, you mention something about the “increased presence of ER,” but there is nothing in your analysis related to changes in the ER. Or am I missing something?
- The last paragraph: “We hypothesize that this mismatch in information transfer at a crucial hippocampal tripartite synapse may contribute to higher-level cognitive deficits associated with AD.” This requires further elaboration. It does not mean anything. Please provide a reference, and make the reader understand how and why.

General grammar notes.

- Please be careful: many times, you use “through”/“from”/” via” instead of “by;” and “for” instead of “of.”
- There are also ambiguous terms: “low endogenous Ca2+ buffering:” low what?
- The first paragraph of “Gliotransmitter release” needs rephrasing.
- … mobile vesicles that are ‘not’ localized in close confined spaces … also, I do not understand the need for quotes around ‘not.’ There is a typo in Syt7 in the following sentence.
- p14: the first sentence of “Modeling vesicular release at a single astrocytic process” can be dropped: i.e. “Despite several… not present.” No need for a new line after the first paragraph.
- p15: “perfectly match” → match; arise→ originate; discrete→ distinct?
- p17: two critical regulators of astrocytic calcium signaling changed by AD.
- Lastly → finally.
- p21: “difficulty”: you mean the current technological limits? You should provide a reference at the end of this sentence. “Apart from … Ca2+ release events” is hard to understand: “While both” → “Although our results are in agreement with experimental studies…” What do you mean by “temporal relationship”?
- p22: All AD groups displayed lower values than control – lower values of what?
- p23: To address this gap → to fill in the gap… for astrocytic calcium signaling and the downstream gliotransmitter release…
- p26: underscores → “likely underpins”. Go astray → this is lost; Ab-induced alterations with astrocytic Ca2+ → alterations of what? Drop “at a single process level”. “abnormal Ca2+” where is this evidence?
