# Supplementary material for: Amyloid pathology disrupts gliotransmitter release in astrocytes
Source: PLoS Comput Biol. 2022 Aug 1;18(8):e1010334. doi: 10.1371/journal.pcbi.1010334 (PMC9371304; doi:10.1371/journal.pcbi.1010334)
Supplement: Attachment — Submitted filename: PCOMPBIOL-D-20-01194R1.pdf [file pcbi.1010334.s012.pdf]

PCOMPBIOL-D-20-01194R1
Title: Amyloid pathology disrupts gliotransmitter release in astrocytes
Authors: Anup G. Pillai and Suhita Nadkarni

Overview

I appreciate the effort put by the authors in improving their manuscript with respect to the original submission. However, while I found improved figures and Result's exposition, I am dismayed by the pervasive sense of carelessness still emerging from the writing style. Again, Citations appear to be often made superficially and lack a clear rationale with the cited work. The logical flow in crucial sections like Introduction and Discussion remains fragmented. This is nerving. Very often, when I read your new manuscript, I came to wonder if you were aware of what you mean in English or not since, on several occasions, your sentences do not make any sense, and your reasoning is flawed. We are reaching a stage in the computational glioscience literature where we are in charge of keeping the field rigorous and prioritizing quality and scientific rigor over bombastic statements and lousy material. While your editing would go towards the former, what still emerges from your manuscript is the latter. I am once more asking for major editings. The hope is that, since the first round took you about a year to be addressed with debatable results in terms of quality, this second occasion could instead be managed more effectively with the maximal outcome: i.e., we could finally go into publication. If not, frankly, my level of frustration with this work is reaching saturation.

On a side note, on this round, not only do you not provide a manuscript with numbered lines making exact referencing to parts of the text impossible, but also you do not number pages, making the whole reviewing effort an even more daunting task. Thank you for showing respect and appreciation for your reviewers' time and effort. In your response to the reviewers and me, I also appreciated how you replied to several points by literally pasting the same answer.

Suggestions for detailed corrections

Title
Your title is emblematic of my above remarks. "Amyloid pathology disrupts gliotransmitter release in astrocytes" is arguable on many levels. First, "Amyloid pathology" does not mean anything. Amyloids can be several, and you are considering only beta-amyloids. Pathology is not necessarily associated with amyloids. "Disruption" is imprecise since it implies deviation from a norm that is not clear. "Gliotransmitter release in astrocytes" is almost tautological, besides the fact that it should be gliotransmitter release *from* astrocytes. I would frankly advise editing the title along the lines of something more specific of your study: "Amyloid-beta decorrelate calcium dynamics from gliotransmission in astrocytes."

The title page in the Acknowledgements: "and *by the* Indian... and Research *at* Pune."

Abstract

I would advise rephrasing along the following lines:
Extracellular deposition of amyloid-beta (A\beta) is recognized to correlate with synaptic dysfunction and alteration of astrocytic signaling, but the biophysical mechanisms underpinning such alteration remain elusive. On the other hand, astrocytes are known to be active modulators of synaptic function through the regulated release of neuroactive molecules (or gliotransmitters) in a calcium-dependent fashion. We introduce a biophysical model for astrocytic perisynaptic domains to characterize gliotransmission release by A\beta-dependent modulation of astrocytic calcium dynamics through plasma-membrane pumps (PMCA) and metabotropic glutamate receptors (mGluRs). Our model realistically captures astrocytic hyperactivity in the presence of A\beta-related pathologies. At the same time, it also predicts faster rates of gliotransmitter depletion that ultimately decorrelate gliotransmitter release from underpinning calcium dynamics, with the possibility to disrupt the delicate control of synaptic function by astrocytes.

Authors' Summary

The first sentence is incorrect. Again: the fact that astrocytic signaling is critical for information processing at synapses is not proven; it is just speculated. "Aberration" is not the right word. The signaling that you consider – gliotransmission – has only little been linked with AD. The further reference to technological limitations comes out of the blue since you don't address this aspect in your work, and it is also not the main point of your study. Conclusions: rewrite it as a simplified version of the Abstract just for the lay public.

Introduction

There are three main themes in your work: Amyloid-beta, astrocytic calcium signaling, and gliotransmission. You are linking Abeta with calcium signaling and calcium signaling with gliotransmission, but what I am missing to close the logical loop is why we should care about gliotransmission in the context of amyloid-beta—mediated pathologies. You do not provide a structured, logical flow to account for these three factors. This is not only at the Introduction level but also in the presentation of the model, exposition of Results, and arguably in the Discussion. It should not be that difficult: it is only a matter of linking three concepts. The way you currently present your thoughts is that because healthy synaptic function relies on gliotransmission, which is not a sound argument, then Alzheimer's disease, which involves synaptic dysfunction, could be caused by gliotransmission. You see that the cause-effect logic is not consistent. It would help if you based your rationale on a sound syllogism.

A related issue is that A\beta links with Alzheimer's disease, and you often interchangeably use A\beta and AD. I would advise focusing your work on A\beta (as I suggested in the Abstract above) and then motivating your interest in A\beta instead by its underpinnings of Alzheimer's disease. This perspective could also offer you the chance to elaborate your Discussion better, emphasizing the translational implications of your work.

Based on such considerations, I would reshape Introduction, starting from the general notion of neuron-glial interactions at synaptic loci along the following lines:

Healthy brain function relies on an intricate interaction between neurons and glial cells at synapses [REF]. <Explain how and the gliotransmitter hypothesis. In doing so, talk about gliotransmission, and then emphasize calcium signaling as the crucial mediator of the phenomenon> (should mention Marchaland's work since this is an essential paper in your work).

<Then use calcium signaling and its pathological behavior – describe 'pathological' – based on Kuchiblota et al.'s study, to link to pathology and A\beta, with emphasis on the extracellular compartment>.

<Finally, elaborate A\beta in the context of AD, and motivate your study accordingly.>
<You should also include a paragraph that describes state of the art on models of gliotransmission and the computational need for your model>.

In your current version:
Alzheimer's disease (AD) is a ~~highly debilitating~~ *prominent* neurodegenerative ... [1]. According to the amyloid-*beta* cascade hypothesis~~, one of the most prevalent neuro-centric theories for AD-related dementia,~~ amyloid-beta accumulation *in the extracellular space* is the pathological hallmark of AD, and synaptic dysfunction [2] <Note here that synaptic dysfunction is another concept. Your reasoning has a gap.> ~~However,~~ *R*ecent... [5,6] <Panatier did not support this argument; Savtchouck's work is on NMDARs related to gliotransmission... these are not wrong references.> It is now widely accepted (maybe YOU are widely accepting, not me or others) that optimal <who ever spoke even of optimality principles?> interaction between neurons and astrocytes is crucial for normal brain functioning [7-11] <Only ref. 8 could be pertinent here, yet it is not even the most appropriate since it deals with gliotransmission only. You need more general work: probably Khakh and Sofroniew NRN 2016, or maybe the Kastaneka et al.'s roadmap paper in Glia 2016.>

... In vivo observations of elevated ... In this study, we focus on two crucial calcium signaling mechanisms... Apart from elevated ... However there is little understanding... The whole logical flow here is broken—no clear cause-effect relation. Different concepts are coming into place. Lousy.

... In high levels on astrocytic compartments... Moreover, we consider <use list>: (i) the stochastic gating of IP3Rs; and (ii) different molecular mechanisms for gliotransmission, respectively by kiss-and-run vs. full-fusion of gliotransmitter-containing vesicles [11, ~~25~~ Replace by De Pitta et al., *Neurosci.* 2016].

Drop "Taking into account these molecular... [8,9,11,23]

Methods

What I found disturbing, to say the least, is that you talk about spatially-related quantities "IP3R clusters," "vesicle movement," or "trafficking," and so on, when your model does not include space at all. These terms would make sense only if you were dealing with PDE models. But you are just dealing with lumped compartments. Avoid such ambiguity, please.

The whole introductory part of Methods is not informative unless you describe the essence of your model by listing all the variables as:

dC_cyt/dt = f_1(c,I,G...)
dI/dt = f_2(C_cyt, I, h...)
dS/dt = ...
...

Otherwise, drop it.
Likewise, I would drop, whenever possible, details on parameter choices (as these are detailed in the supplementary tables). Moreover, what is this use of uncommon abbreviations like "conc:" what do you think you are doing? Writing a shopping list?

Your notation also is not consistent. Sometimes you use K_D, other times k_d, other times K_d. Please be coherent.

- "... cluster of 5 IP3Rs:" you see? You cannot have a cluster in a lumped parameter model.
- "The model reliably captured stochastic Ca2+ transient through the IP3R cluster using a Langevin approximation of the Li-Rinzel IP3R model developed by Shuai et al. [43]." What is "reliably" for you? There is no mention of the IP3R cluster in Shuai's original work unless you go to the PDE version of that model. It is not a Langevin approximation (as I pointed out previously). The LR model for IP3R is not correct. Li and Rinzel provided a reduction of the DYK model. Please be careful with these details.
- "... A passive... is not fully understood [not relevant, but if you keep it then a REF is needed].
- Glutamate stimulation... [44] is a wrong reference.
- Eq. 4: Please use conventional symbols: V_max, \kappa_d, coeff-->n. And correct the main text accordingly,
- Eqs. 6 and 9: you are using the same symbol "r" for different things.
- The respons~~ive~~ ~~measure~~ *r* was computed by ... T_0 and T_stim (use the same case for quantities that bear the same dimension).
- Flip eq. 8 with eq. 9. Move "Asynchrony value of 1 corresponds to..." after equation 9.
- "Synchrony was computed for each Ca2+ interevent interval by a matrix of event timings vs. trials following the treatment originally introduced by Pinsky and Rinzel [56]. At each trial j... from now on to eq. 7 whatever you wrote is unreadable.
- Computation of calcium event features. Full width at half maximum (FWHM) as the time interval between left and right halves of a calcium peak. For rise times, we considered the time

interval between... Why do you keep using "horizontal distance"?!? Geese, say things for what they are.

- C++ (and not C^{++}) we compiled by GCC7.5 (welcome: we are now at version 20+). "institution" what Institution? A Hospital? Or perhaps an Academic Institute? Which one?

Results

- Organize your Results in two-three sections: Calcium/gliotransmission model; Ab dependence... and so on.
- The PMCA pumps ~~present~~ on the plasma membrane mediate... within a process *mainly* arise *by* stochastic ... The dynamics of these Ca2+ spikes is also ...
- At some point at p15 (if I counted it right...) "Both fast kiss-and-run-like confined releases and slow-spreading... full-fusion in astrocytes." This whole paragraph pertains to modeling methods rather than to results.
- On p16: "Similar to a previous study [23]" It is still Marchaland et al. Your phrasing does not make any sense.
- On p18, suddenly out of the blue, you introduce PMCA functionality: "Multiple studies have reported..." It looks like you need to start a new section of Results.
- Soon after on p19 you start talking in terms of astrocytic groups with Ab pathology. Please DO NOT. You are not modeling astrocyte groups. You are simulating different conditions. Likewise, do not use terms such as Ab-groups. Stick to meaningful and transparent definitions such as "Ab conditions," "astrocyte domains" in different conditions, and so on...
- on p19 Refs 68-70 are not in pathological conditions; hence they are not appropriate.
- Abeta pathology enhances gliotransmitter release events: Start directly from "We next describe the impact of..."
- "Abeta induces frequency-dependent modulation of Ca2+ event synchrony: synchronous discharges... and pathological roles [add REF]." What the hell are you talking about? "Discharges"?!? Discharge is related to electrical quantities, maybe, but not to chemical signals as in astrocytes. Mind your lexicon. Furthermore: synchronicity requires two terms: what are they?
- ... p22: There is accumulating evidence that ... Ca2+ and/or gliotransmitter release events boosts... Therefore we next investigated... we computed temporal synchrony at different inter-event intervals (see Methods) to study... (Figure 7A).
- p24. We quantified... -> As expected, the cross-correlation between Ca2+ peaks and gliotransmitter release (see Methods) was high...
- p25: ... loss of temporal correlation between calcium and release events in different Ab conditions with respect to physiological ones.

Discussion

- Abeta, astrocytic Ca2+ signaling, and gliotransmission have remained unclear. Here gliotransmission seems disconnected from the other concepts. Hopefully, by the above suggestions, you will be able to rephrase coherently.
- Our results quantitatively describe... Our model can quantitatively reproduce experimental observations for altered astrocytic calcium spiking statistics mediated in the presence of Ab pathology. Specifically, we pinpoint that such alterations could critically depend on Ab-mediated regulation of mGluR <what> and PMCA <what>.
- ....We build on previous models of intracellular calcium signaling to seek an accurate description of synaptically-activated gliotransmission at individual perisynaptic astrocytic processes. Our description includes...
- p26 Ca2+ events from astrocytic microdomains... a broad range of kinetics...there is no "kinetics" for Ca2+ signals, rather only "dynamics." [30,39,40] these are incorrect. Drop 39 and 40. Add instead Panatier et al., 2011; Bindocci et al., 2017.
- p25 "Despite... Ca2+-dependent vesicular release [78,79]." Unrelated. Drop.
- P27 When AD-mediated changes are implemented in our model... > When we considered AD-mediated changes of astrocytic signaling, our model predicts that... consistently with experimental data [13]. Additionally, and in agreement with in vivo findings,...
- "An important prediction of our model is that this change... Ab-PMCA groups" Come on, this is not a prediction. It is evident from your model. Drop it.
- P28: in striking agreement with the model prediction... what are you talking about? This is speculation. There is no agreement whatsoever. Rephrase.
- P29: While in vivo studies confirm synchronous Ca2+ activity ... (synchronous w.r.t to what?) ... Evidently, despite the high rate of Ca2+... <I missing the logical link>...
- Taken together, it is worth noting that in our simulations, the effect of Abeta is most potent at 0.4-10Hz... so what? Why is it worth noting? What is the logical link with what you told me before?
- ... To conclude, we presented a biophysically detailed model to realistically simulate ... to verify our modeling assumptions and provide valuable insights on the role of gliotransmitter release under normal and pathological conditions <again: gliotransmitter release is only marginally discussed in the context of Abeta pathology at this stage>.

Figures

You cite Figure 2 before Figure 1, and several references to Figures are incorrect (e.g., Figure 3 in Discussion is not the right figure).

Figure 1. A. Since you are considering glutamate spillover, you should sketch some synaptic structures near your synaptic process. B. What are the different colors? Very confusing, frankly. Just show traces in one color. Use dashed lines for DHPG application as elsewhere. D–G Probably, it is better to show these data by histograms rather than piecewise linear curves. F. Use conventional labels: FWHM (ms).

Figure 3. Make D–F plots of the same size of A–C, and vertically align their y-axes to these latter. Here and in the remainder of the figures, they are rendered the same in grayscale if you use blue and red. Use color-blind colors, please: e.g., orange and green/blue and so on. Why not put DHPG on top of all graphs (also in Figure 1) to have the axes start from 0 and improve readability? B and C. Make error bars thinner and data points larger. D-E, what's the point of showing errorbars/data points if they are within the thickness of the line? Tune line widths in a meaningful fashion, please.

Figure 4D. Replot as 2D heatmaps color-coded for calcium concentration. Use the same color scale for all the plots. Alternatively, use the color scheme (after correcting it for blinds) to avoid using titles as x-axes labels. Plot all figures in a standard fashion: no tiny axes vs. full axes: only full axes. Hence show in D trials and time for all panels.

Figure 5. A. Same considerations as in Figure 4D. B–F Please add Ticks and Y-label to right panels. Correct for the color blind.

Figure 6A. Drop y-axes between panels except for the leftmost one. But specify ticks and x labels for all panels. Use at least 3-4 x ticks.

Figures 7A,C. Repeat ticks and labels for x and y axes, please. What's this choice of non-equispaced ticks on color bars and x-axes? Change it, please. On the y-axes, also, please adopt ticks with the same number of decimal figures. Up to 4-5 ticks in this case.

Supplementary Material / Appendix 1

Again, you do not use the consistent notation: Glu is sometimes denoted as a concentration, i.e. "[Glu]," some other times as a "Glu." Please be consistent. J_IP3P should have been corrected by now and be instead J_IP5P or J_5P. Moreover, make sure to number your equations. q_2 should be rather Q_2 for historical reasons. Nonconventional symbols for multiplication "x" (again!): please drop them. What's the point of showing J_syt4/7 if we do not have the equations for S_i / Y_i? Would you please show these equations? The equation for glutamate degradation cannot be written as it is: you need to justify why you do not consider the contribution to degradation by binding by receptors. Also, what is the source of extracellular glutamate? I am missing your "stimulus(t)" equation.

Since you deliberately mention Hill functions in your main text, use the classic notation for such functions then: H_n(x, K), to simplify your equations.
