# Supplementary material for: Amyloid pathology disrupts gliotransmitter release in astrocytes
Source: PLoS Comput Biol. 2022 Aug 1;18(8):e1010334. doi: 10.1371/journal.pcbi.1010334 (PMC9371304; doi:10.1371/journal.pcbi.1010334)
Supplement: Attachment — Submitted filename: Pillai_et_al_2021_response_to_reviewers.docx [file pcbi.1010334.s010.docx]

**Reviewer #1**

*Pillai and Nadkarni introduce a novel model of astrocyte-synapse interactions that revolves around the biophysical description of calcium-dependent glutamate release from astrocytes. In particular, they distinguish between different mechanisms of glutamate exocytosis – kiss-and-run vs. full-fusion – by considering the Ca2+-dependent dynamics of different synaptotagmins. Then, they proceed to mimic amyloid-like pathology changing values to model parameters, and documenting, through numerical simulations, how amyloid-beta could affect astrocytic physiology, and astrocytic glutamate release in particular. The study is original and of interest to PLoS Computational Biology, with the potential to reach out to a broad audience, comprising both experimentalists and computationalists. Nonetheless, there is ample space for improvement of the manuscript in its present version. There are at least three main issues with the present paper. First, the research question is not clear, and the way the material is exposed is not exhaustive and somehow lousy. Second, there is a critical issue with the references cited by the authors, which often do not support the authors’ claims. Third, the writing style is poor, making the reading of the manuscript hard for several broken sentences, ambiguous terminology, and logical flow that is not consistent. For these reasons, I am advising for major revisions, which I am detailing in the attachment.*

**A:** We thank the reviewer for finding our study interesting and original. We have made extensive changes to the manuscript to address all the concerns by the reviewers. We believe the manuscript is now ready for publication.

***C1****.* *You are missing to explain to the reader in the Introduction why we should care about gliotransmitter release from the astrocyte in the context of Alzheimer’s. In doing so, there is a mismatch between Introduction and discussion, which should be resolved accordingly.*

**A**: We thank the reviewer for commenting on this issue. Both the discussion and the introduction include discussion (with relevant references) on the link between gliotransmitter release and Alzheimer’s pathology in the new version.

***C2****. Refs. 1–4 are not proper. You need reviews here. And, as much as possible, from the past decade, that is, not older than 2010, please.*

**A**: We thank the reviewer for pointing this to us. We have updated the references to more recent reviews on astrocytic calcium signaling (Zorec et al., 2012; Araque et al., 2014; Volterra et al., 2014; Bazargani and Attwell, 2016; Savtchouk and Volterra, 2018).

***C3****. Ref. 5 is not about gliotransmitter release but discusses the sources of variability of calcium in astrocytes. Also, why are you limiting your reasoning to the hippocampus at this stage?*

**A**: We apologize for this oversight. We have now corrected the reference. We agree with the reviewer's suggestions that the discussion should not be limited to the hippocampus and have made the changes in the manuscript accordingly.

***C4****. Ref. 10 is a bit old an should be complemented by Bazarghani and Attwell, Nature Neurosci. 2016. Similarly, when you say “fast, high-amplitude and highly compartmentalized Ca2+ signals,” references 11 and 12 are not correct. Those works did not focus on calcium signals. Moreover, when talking about calcium compartmentalization, those references are still not appropriate. Again, use Bazarghani and Attwell and references within to properly support your claim.*

**A**: We have added two additional references to strengthen our statement on astrocytic Ca^2+^ excitability and signaling (Parpura et al., 2011; Zorec et al., 2012; Bazargani and Attwell, 2016). We have also cited two other studies on fast Ca^2+^ transients from astrocytic processes (Di Castro et al., 2011; Tang et al., 2015; Bazargani and Attwell, 2016; Rungta et al., 2016).

***C5****. Since most of your modeling work relies on Ref. 12, it is probably worth explaining it more. However, linking calcium machinery and the existence of different exocytotic mechanisms by mGluRs and ER clustering is not by Refs. 12 and 13. Instead, the way to do it is to look into the last part of Ref. 5 and take into account the recent work by Volterra and Savtchouk in J. Neurosci 2018.*

**A**: We agree with the reviewer that we should have described the work by Marchaland et al. in more detail. In the new version of the manuscript, we have the appropriate additions. We are grateful to the reviewer for suggesting additional relevant references on astrocytic calcium signaling and release machinery.

***C6****. In the presentation of Synaptotagmins, why should we care about Syt1? I assume because this is the main one involved in synaptic neurotransmitter release, but then you should state it and provide essential references.*

**A**: We thank the reviewer for highlighting this fact. We compared *Sy4* to *Syt1* because although *Syt4* lack the functional C_2_A domain (Thomas et al., 1999), it promotes rapid exocytosis from the binding of Ca^2+^ to its C_2_B domain. Indeed, *Syt4* can substitute *Syt1* in the presynaptic terminal (Robinson and Ranjan, 2002). We have updated the sentence to explain our reasons better and cited the relevant references (More details are also available in S3 Appendix).

**C7**. *Ref. 17 is in cerebellar astrocytes: not what you want if you are considering the hippocampus.*

**A:** We agree with the reviewer that our reference for *Syt7* (Wolfes et al., 2017) is a study on stellate astrocytes and have clarified this in the new version of the manuscript. Our model is built under the assumption that release machinery is conserved in astrocytes. Whether we will need to make improvements to the release machinery in future versions of the model will be clear when data on the hippocampus is available. Interestingly, Ref 17. does mention stellate morphology, but it is not clear if these are from astrocytes are from the cerebellum alone (the cultures were prepared from cortices extracted from E19 embryos). Additionally, stellate astrocytes are also present in the hippocampus (Carvalho-Paulo et al., 2018).

***C8****. “Equipped with this elaborate machinery for exocytosis that is analogous to the presynaptic terminal in neurons” – a reference is missing here.*

**A**: We thank the reviewer for bringing up this oversight. We have modified the sentence and added the appropriate citations.

***C9****. There is ambiguity in your exposition of astrocyte-mediated synchrony. Ref. 19 deals with SICs and looks at spontaneous calcium events rather than evoked ones; hence it is not appropriate. Also, it is not clear whether SICs, and neuronal synchrony thereby, is by exocytosis of glutamate by astrocytes. Finally, in your results, you are looking at the synchronization of glutamate release with upstream neural activity, and not, as the studies that you refer to point to, to synchronization of downstream neuronal firing with respect to calcium-dependent SICs/glutamate release. Be careful: the field is evolving rapidly, and these details are crucial nowadays with respect to 10 years ago.*

**A**: In agreement with the reviewer's suggestion, we have modified the questionable sentence and cited two studies that demonstrate the modulation of pre- and postsynaptic activity by astrocytic gliotransmitter release.

1.	Savtchouk I, Di Castro MA, Ali R, Stubbe H, Luján R, Volterra A. Circuit-specific control of the medial entorhinal inputs to the dentate gyrus by atypical presynaptic NMDARs activated by astrocytes. Proc Natl Acad Sci U S A. 2019. doi:10.1073/pnas.1816013116

2.	Jourdain P, Bergersen LH, Bhaukaurally K, Bezzi P, Santello M, Domercq M, et al. Glutamate exocytosis from astrocytes controls synaptic strength. Nat Neurosci. 2007;10: 331–339. doi:10.1038/nn1849

***C10****. The sentence associated with Refs. 21,22 can be dropped.*

**A**: We agree with the reviewer and removed this sentence from the revised manuscript.

***C11****. Ref. 28 is not related to any aspect of pathology. You should look instead into work by Molofsky or Khakh for more appropriate work.*

**A**: We agree with the reviewer that the study by Perea et al. (Perea, 2005) may not be the most appropriate in this context in the manuscript. We, therefore, cite the reviews from Adele Vincent (Vincent et al., 2010) and Alexei Verkhratsky (Verkhratsky et al., 2017) that nicely elaborate on several astrocytic mechanisms including Ca^2+^ signaling and gliotransmitter release in AD.

***C12****. Ref 29 is also not appropriate. You should refer here to the original studies that identified Orai1 and STIM1 channels.*

**A**: We thank the reviewer for bringing this to our notice. We have added citations to the seminal works (Liou et al., 2005; Roos et al., 2005; Vig et al., 2006) that identified the two crucial molecular components (STIM 1 and Orai1) of SOCs that are involved in the replenishment of ER calcium levels (see below).

“Over longer timescales, in response to emptying of ER, subsequent entry of calcium takes place by store-operated Ca^2+^ release-activated Ca^2+^ channels encoded by Orai1 and STIM1 (Liou et al., 2005; Roos et al., 2005; Vig et al., 2006). However, these timescales are beyond the scope of the present study and therefore not included in the present model.”

***C13****. Ref. 31: why should we consider a study in neurons, if you are reasoning on astrocytes?*

**A**: Following the reviewer's suggestion, we now refer to studies (Marchaland et al., 2008; Santello et al., 2011; Srinivasan et al., 2015a) that specifically report on IP_3_ receptor-dependent Ca^2+^ transients in astrocytic microdomains (see below).

“The parameters of cytosolic Ca^2+^ buffers (conc: 50 μM; K_D_: 20 μM) were adjusted to reproduce the fast kinetics of mGluR-dependent Ca^2+^ transients in processes as reported by previous studies (Marchaland et al., 2008; Santello et al., 2011; Srinivasan et al., 2015b).”

***C14****. Refs. 26,34 following “As a result, ER resting Ca2+ levels…” are not representative of previous models: the literature on calcium signaling is vast, and either you refer to relevant reviews here, or you look into studies that you directly use/extend in your modeling work.*

**A**. We agree that this sentence is an overstatement. We apologize for it. We have removed the sentence in question in the new version.

***C15****. When discussing Ref. 34, note that you are not using a “Langevin approximation” – there is no such thing. Instead, you are using a Langevin description for stochastic IP3R gating.*

**A:** We thank the reviewer for pointing this out. This is corrected in the revised manuscript.

***C16****. When discussing IP3 dynamics, following the description of PLC-delta, IP3-3K, and IP-5P modeling require the citation of Matrosov et al., Computational Glycoscience 2019.*

**A**: As suggested by the reviewer, we have cited the recent work by Matrosov et. al. (Matrosov et al., 2019). Please refer to the sentence below.

“We incorporated two IP_3_ degradation pathways in the model, namely, inositol polyphosphate 5-phosphatase and IP_3_ 3-kinase (De Pittà et al., 2009; Matrosov et al., 2019). We approximated the above slow IP_3_ regulating mechanisms with four separate Hill equations (S1 Appendix & S1 Table).”

***C17****. Ref. 36 should also be complemented by the more recent work by those investigators in Computational Glioscience 2019. This latter reference and the citations within will likely tell you why a statement such as “Ca2+-independent IP-5P-mediated degradation” is not correct.*

**A**: As per the reviewer's suggestion, we have now cited the recent work by De Pittà et al., in the revised version of the manuscript. We also thank the reviewer for bringing to our attention the dependence of IP_3_ 5-phosphatase activity on both Ca^2+^ and IP_3_. However, for simplicity, in this study, we model IP-5-phosphatase activity with a linear dependence on IP_3_ as been described before in previous studies (De Pittà et al., 2009; Matrosov et al., 2019).

***C18****. Ref. 12 about vesicle distribution and kinetics in the first paragraph of the section “Gliotransmitter Release” is not appropriate. Arguably, works by Zorec and Parpura should be cited instead. The same Ref. is inappropriate at the end of the first sentence on page 9.*

**A**: We thank the reviewer for bringing this to our notice. We have updated the manuscript citations accordingly.

***C19****. Ref. 44 on page 11 is not sufficient. You should refer to De Pitta in Computational Glioscience 2019 to accurately estimate the concentration range of Ca2+ threshold for gliotransmitter exocytosis.*

**A**: We thank the reviewer for bringing to our attention the excellent theoretical exposition of gliotransmitter release by De Pitta in Computational Glioscience 2019. We now cite this work in the revised version of the manuscript.

Our choice of Ca^2+^ threshold (200 nM) was based on a previous study that reported on the physiological minimum to evoke a reliable glutamate release from astrocytes (Parpura and Haydon, 2000). Moving the threshold slightly above or below did not alter the results appreciably as most events have much higher Ca^2+^ amplitudes with a mean value close to 2.4 µM. Interestingly, with this set value of Ca^2+^ threshold we obtained a very close match between the model and experimental results from Marchaland et. al. (Marchaland et al., 2008).

***C20****. Refs. 50 and 51 (p15) – I am missing the logical link: I do not see how these two references are related to your current work. The whole sentence they refer to should be moved to Methods.*

**A**: We apologize for this oversight. These references have been removed from the revised manuscript.

***C21****. Ref 43 is not appropriate for the temporal profile of extracellular glutamate. For example, you should refer to other studies by Clements et al., (1992-1996); Rusakov and co. (2000-2004) or, more recently, De Pitta in Computational Glioscience 2019).*

**A**: We have added the below sentence as well as added the following references for glutamate profile in the revised manuscript.

“Peak amplitude and decay of glutamate (**S1 Figure**) were close to experimentally reported timecourse of spillover glutamate around an astrocytic process (Clements et al., 1992; Diamond, 2005; Hires et al., 2008).”

1.	Diamond JS. Deriving the glutamate clearance time course from transporter currents in CA1 hippocampal astrocytes: Transmitter uptake gets faster during development. J Neurosci. 2005. doi:10.1523/JNEUROSCI.5125-04.2005

2.	Hires SA, Zhu Y, Tsien RY. Optical measurement of synaptic glutamate spillover and reuptake by linker optimized glutamate-sensitive fluorescent reporters. Proc Natl Acad Sci U S A. 2008. doi:10.1073/pnas.0712008105

3.	Clements J, Lester R, Tong G, Jahr C, Westbrook G. The time course of glutamate in the synaptic cleft. Science (80- ). 1992;258: 1498–1501. doi:10.1126/science.1359647

***C22****. Ref.28 on p21 is not appropriate. A Review by Perea and co-workers could instead be more reasonable. Or work by Savtchouk and Volterra, JN 2018; De Pitta et al., 2015; Araque et al., 2014. The sense of the sentence associated with this reference is also wrong. Astrocytes modulate synaptic transmission, and this can result in neuronal synchrony, and not the opposite. If you are looking at independent mechanisms, then gliotransmission is not the common pathway. You will need to look into ion homeostasis (see for example, work by Nedergaard’s group).*

`

**A**: We agree that that the sentence is confusing. We have updated the sentence to better explain our motivation to examine the temporal correlation between gliotransmitter release and astrocytic calcium activity.

“Temporal correlation between Ca^2+^ and gliotransmitter events is critical for astrocytic modulation of synaptic activity at short timescales (Jourdain et al., 2007; Wallach et al., 2014; Savtchouk et al., 2019). We have demonstrated that Aβ pathology modulates both Ca^2+^ signaling and gliotransmission in a stimulation-dependent manner (Figures 5 & 6). Additionally, gliotransmitter release also depends on the availability of vesicles and the Ca^2+^ affinities and kinetics of the two synaptotagmins. Each of these can potentially be altered by Aβ-induced changes in mGluR and PMCA functionality.”

“An important prediction from our study is the disruption of the temporal correlation between Ca^2+^ and gliotransmission by Aβ mechanisms. As astrocytes are electrically silent, the tight temporal correlation of Ca^2+^ events with release is critical for astrocytic feedback (Calì and Bezzi, 2010). While the biological underpinnings of asynchronous release are unclear, it has been suggested asynchronous release in interneurons serves to desynchronize the activity of downstream neurons (Hestrin and Galarreta, 2005). Recent evidence establishes astrocytes as a strong modulator of brain waves (Foley et al., 2017; Buskila et al., 2019). It is interesting to note that in our simulations the effect of Aβ is strongest at the low-mid stimulation regime (0.4-10Hz) where the temporal correlation is high in the control group. Although may not be directly related, delta (2-4Hz), theta (4-8Hz) and alpha (8-13Hz) waves are strong bio markers for early detection of cognitive abnormalities related to AD (Vecchio et al., 2011).”

***C23****. Ref. 63 is Ref 12.*

**A**: We apologize for the mistake. We have removed this reference duplication in the revised manuscript

***C24****. Refs. 19,65 on p24 are not appropriate. Angulo’s work is not on calcium waves, and synchronization is not the main result. Lavrentovich’s study instead is a straightforward one on calcium waves, but not in a biophysical way as yours. Also, your model does not account for calcium waves.*

**A**: We apologize for the mismatch between the sentence and the citations. We have corrected the sentence in the revised version.

***C25****. Refs. 12,66 on the second row on p25 are not appropriate. Microdomains in vitro are not relevant. Marchaland’s work was not on microdomains. Covelho’s work was on different types of gliotransmitter release from the same astrocyte. Di Castro’s work is appropriate but should be cited along with Panatier’s companion paper in Neuron 2011.*

**A**: We agree with the reviewer that Panatier's work on Ca^2+^ signals from individual microdomains on astrocytic processes is more relevant to our work. The revised manuscript now cites this important study. Additionally, both Di Castro’s and Panatier’s studies also use *in vitro* preparations (brain slice) (Di Castro et al., 2011; Panatier et al., 2011). We cited the study by Marchaland et al., as they clearly described the fast (millisecond) timescales of Ca^2+^ transients and exocytosis (Marchaland et al., 2008); from their abstract "Our main findings indicate that (1) exo-endocytosis in astrocytes proceeds with a time course on the millisecond time scale ... and (2) exocytosis is controlled by local Ca^2+^ microdomains". There is also a close match in the ROI area (<= 4 μm^2^) in Marchaland’s study with the mean microdomain length (1.5 ± 0.1 μm) reported by Panatier et. al., (Panatier et al., 2011).

***C26****. Ref. 4 on p25 for the structural information on the mobile vesicle pool is not appropriate. Jourdain’s work was not dealing with any characterization of such kind, and they never proved the existence of a vesicle pool – this is, in fact, an open question in the field, and an ansatz in your modeling work.*

**A**: We agree with the reviewer that detailed studies on astrocytic vesicles’ dynamics within a single compartment are yet to be done. The EM study by Jourdain et al., 2007 was one of the first to report on the presence of glutamate containing vesicles as far as 200 μm from the plasma membrane. Other studies (Potokar et al., 2005; Crippa et al., 2006; Stenovec et al., 2007) have reported the presence of mobile vesicles. These studies provide indirect evidence to our release model with docked and mobile vesicles that are distinctly targeted by the fast (*Syt4*) and the slow (*Syt7*) synaptotagmins, respectively. We have stated the basis of our release model clearly in the revised manuscript (S3 Appendix).

Below is an excerpt from the revised manuscript that discusses these aspects.

“An important functional consequence of astrocytic Ca^2+^ signaling is Ca^2+^-dependent vesicular release. Multiple evidence supports our model for gliotransmission with two synaptotagmins that have different kinetics and Ca^2+^ affinities (details are in **S3 Appendix**) (Rao et al., 2004; Zhang et al., 2004). Model also includes docked and mobile vesicles that are targeted by *Syt4* and *Syt7*, respectively (Crippa et al., 2006; Marchaland et al., 2008; Liu et al., 2011). Our choice of parameters are from experimental measurements that have reported kiss-and-run and full fusions exocytosis with distinct timescales of exocytosis, endocytosis, and reacidification rates (Bowser and Khakh, 2007; Marchaland et al., 2008). Notably, *Syt4* is exclusively activated by fast and high amplitude Ca^2+^ events and cause a fast synchronous release of docked vesicles via kiss-and-run mode. Such Ca^2+^ events have been observed from astrocytic microdomains with ER tubules around docked vesicles to cause rapid kiss-and-run release (Marchaland et al., 2008). On the other hand, *Syt7,* whose Ca^2+^ affinity is high is activated by low amplitude and slow Ca^2+^ events and cause slow asynchronous release of mobile vesicles through full fusion. Not only are mobile vesicles observed in astrocytes, they are also transported in an activity-dependent manner (Stenovec et al., 2007; Potokar et al., 2013). Considerable portions of these vesicles become immobile once they reach the distal end of processes, suggesting vesicle docking (Crippa et al., 2006; Potokar et al., 2009). These and other findings support our model that implements fast synchronous release of docked vesicles and slow, asynchronous release of mobile vesicles (Malarkey and Parpura, 2011; Wong et al., 2015).”

**Model Presentation**

***C27****.* *At the end of the paper, I want to read an appendix that reflects the different subsections of “Methods/Models,” and explains individual model’s modules. Then, you append one single long table, with different modules separated by sections, for all the parameters, and your choice of values, but not equations. Finally, a second appendix, or each of the subsections of the former appendix, should clarify your choice of all model’s parameters / or parameters related to that specific module, providing the necessary references. With this regard, note that the long intermediate paragraph on page 9, for example, is more appropriate within a section that explains how you estimate parameters in your model.*

**A**: We thank the reviewer for the suggestions. We have added three appendices for the paper. In appendix 1 (**S1 Appendix**), we have grouped and numbered all the equations in separate sections. All the parameter values are in the accompanying table (**S1 Table**). In appendix 2 (**S2 Appendix**), we elaborate more on the choice of model parameters for the gliotransmission model and the modifications to parameter values to match the model results with experimental data. In appendix 3 (**S3 Appendix**), we elaborate on the choice of synaptotagmins and their Ca^2+^ affinities.

***C28.*** *Although you refer to your model as a model of the tripartite synapse, in practice, your model is a model of an astrocytic compartment, and glutamate release from there. A further source of ambiguity is that you consider only a single astrocytic compartment, but then, quite often in your simulations, you refer to “astrocytic processes” in a misleading fashion. You do not model many different processes: the source of variability in your simulations comes from the stochastic opening of IP3Rs. Please amend your text accordingly, and try to be as precise as possible, avoiding ambiguities.*

**A**: The reviewer is correct in that we have developed a biophysical model for the activity of a canonical microdomain of an astrocyte. However, by simulating multiple independent trials, we were able capture the variability across astrocytic domains due to stochastic opening of *IP3Rs* that subsequently lead to a variable number of calcium events and stochastic release of gliotransmitters. Nevertheless, we agree with the reviewer that the usage of ‘processes’ could create unnecessary confusion. We have now revised the manuscript accordingly.

***C29.*** *Be careful in your equation 3: there is no such thing as IP3K or IP3P: the correct abbreviations are IP3-3K and IP-5P.*

**A**: We thank the reviewer for pointing this out. Our revised manuscript now stands corrected.

***C30.*** *You introduce an IP3-base in your equation for IP-5P degradation, but are you sure that in your simulations IP3>= IP3-base? If this is not the case, your J_5P will become a production term. Please clarify, and amend your equations accordingly if needed.*

**A**: Baseline IP_3_ in both the control and astrocytic compartments with Aβ pathologies is around 178.8 nM (Figure 4C) which is slightly above the set baseline level of 160 nM. Therefore, IP_3_ 5-phosphatase flux at rest is negative and degrades IP_3_ according to the equation below.

$$\frac{d}{dt}{IP}_{3} = J_{mGluR} - J_{IP3K} - J_{IP3P} + J_{PLC\delta}$$

***C31.*** *On page 9, you write three times “data not shown” on crucial aspects of your model setup. Please, provide these data instead as supplementary figures. This will help to clarify your procedure, ensuring reproducibility.*

**A**: We now provide these data as main or supplementary figures as mentioned below.

1. Decay times of Ca^2+^ events (**Figure 5F**).
2. Temporal profile of glutamate, IP_3_ and Ca^2+^ levels in an astrocytic compartment stimulated with a single glutamate vesicle **(S1 Figure**)
3. Temporal release histograms from astrocytic subdomains stimulated with agonists for different astrocytic receptors (**S2 Figure**). This data was extracted directly from the figures of published experimental studies.

***C32.*** *In the bottom paragraph on page 9, what is “tau”? I do not see any equation that includes it.*

**A:** The tau here refers to 1/*k_Glu_*, the glutamate clearance rate. We have clarified this in the revised manuscript.

***C33.*** *You are making some confusion in the notation. Hill functions, as well as EC50, are used exclusively in chemical/binding reactions. Other formulas that are identical to Hill functions, but are not in terms of chemical equations, are only rational sigmoid functions. This is the case of eq. 5, for example.*

**A**: We fitted the responses with Hill functions to extract a metric (EC50) that captures the shift in Ca^2+^/release event rates from astrocytic compartments with Aβ pathology. We agree with the reviewer that this is a bit confusing. Taking this into consideration, for comparing between groups, we now use mean values at three different stimulation regimes, namely, low (0.4 - 1Hz), intermediate (2 – 10 Hz) and high (20-100Hz).

***C34.*** *Similarly, there is not such a thing as EC50 in Hz. EC50, as the acronym says, stands for halfmaximal “effective concentration.” Please amend your notation in formulas and text accordingly.*

**A**: We have modified our notation in the revised manuscript in agreement with the reviewers suggestion.

***C35.*** *You tend to overuse parentheses. You can easily simplify your notation, dropping parentheses from Eqs. 2, 3, 8, 9. Also, be consistent with your notation, please: if Ca2+ denotes a concentration, then always use [Ca2+], and append as a subscript out of the right bracket the relevant identifier, i.e. [Ca2+]_cyt. Amend accordingly, Eqs. 1,2,4,6.*

**A**: We thank the reviewer for the suggestion. We have updated these equations in the revised manuscript. We also amended all the notations of Ca^2+^ concentrations to be uniform across all equations.

***C36.*** *I do not understand your eq. 6. Can you please use standard notation? What is “÷”? If it is division, use the horizontal bar, and write your formulas by \frac{}{} in the standard form.*

**A**: We apologize for this error. We have corrected equation 6 in the revised manuscript.

***C37.*** *Eq. 8. Use \frac{1}{N_{j,m}}. What about the index of the sum?*

**A**: We apologize for this error. We have corrected equation 8 in the revised version of the manuscript.

***C38.*** *The Section “Computational of correlation between Ca2+ and gliotransmitter release events” could be dropped (or lumped in a Supplementary text). See my comments on “Results and Figures.” If you decide to retain it, explain the several passages by formulas rather than verbally, which will ease its accessibility. Eq. 9 has several typos on the \cdot.*

**A**: The revised version of the manuscript has a detailed description of the algorithm in accordance with suggestion of the reviewer. We have also added new references that cite the original algorithm utilized in our analysis.

***C39.*** *Numerical simulations: You should also lump here Data availability. How is the code implemented? There is no need to reference “Python” but you need to say what version you are using. Instead, you need to cite MATLAB properly as documented on the web, specify the release version, and note that MATLAB is a ®.*

**A**: We thank the reviewer for this suggestion. The new version of the manuscript has the information on appropriate versions of Python and Matlab that were used and link to the data.

***C40.*** *The overall description of docked/fixed, transporter/mobile vesicles’ pools is confusing, since you often exchange the descriptors.*

**A**: We have modified the description of mobile vesicles in view of the reviewer’s suggestion.

**Results and Figures**

***C41.*** *You limit your analysis to a mere description of your simulations’ results, failing to give essential insights on the biophysical mechanisms that, in the model, account for your predictions – and how these predictions reflect in the real brain / AD pathology.*

**A:** We agree with the reviewer that we did not sufficiently explain the implications of the study in our previous version. We have corrected for this shortcoming in the revised manuscript.

***C42.*** *For example, on page 17: when you say that you have increased resting Ca2+ levels but not IP3 levels (Figures 3C – E), so what? Why should we care?*

**A:** We have a new paragraph in the discussion to better connect our predictions to AD pathology.

“In agreement with our finding, enhanced resting Ca^2+^ has been observed in astrocytes from an AD model (Kuchibhotla et al., 2009). However, as our contribution, we predict that this change in resting Ca^2+^ is specific to the decrease in Ca^2+^ binding affinity of PMCA pump (Figures 3B &C). A large fraction of the accumulated Ca^2+^ is sequestered into the ER. Not surprisingly, resting ER Ca^2+^ concentration is also higher in the Aβ-PMCA groups. In line with *in vivo* findings that associates this change in resting Ca^2+^ with synchronous and hyperactive astrocytes, the model predicts high amplitude and frequent Ca^2+^ events in Aβ groups. Astrocytic Ca^2+^ concentration is a critical mechanism for the regulation of blood flow in the arterioles. Astrocytic Ca^2+^ level decreases with excess glutamate in the extracellular space to accommodate the increase in neural activity by an increase in blood flow (Mehina et al., 2017). Lowering of intracellular Ca^2+^ may also reduce astrocytic Ca^2+^ transients and feed forward activation of surrounding neurons through glutamate release. In a striking agreement to the model predictions on dysregulated astrocytic Ca^2+^, both the rigidity and dynamic range of vasculature is compromised in animals displaying excessive amyloid deposition (Kimbrough et al., 2015). These finding, together with model predictions, suggest a prominent role for impaired PMCA signaling in the mediation of amyloid pathology.”

***C43.*** *Another example on p 18: “Both frequency and amplitude of Ca2+ events were enhanced in the AD astrocytic process [note the misuse of “astrocytic process”] compared to the control group. Additionally, we also observed spontaneous Ca2+ events in AD groups with impaired PMCAs” – so what?* *“Notably impaired PMCA activity, apart from increasing spontaneous activity (repetition!), also lowered rise time… – so what? What does this imply in terms of pathology? Why should we care? How do you link these results with experiments?*

*There are many further examples in your results that ask for these questions and remained unanswered. Another important example always on p19: “However, unlike in the AD-mGluR groups, impairment in PMCA functionality was associated with a substantial flattening of kiss-and-run release rates” – ok, but why? What is happening in your model? What are the biophysical underpinnings? p21 “Our results from the model indicate that despite… “so what?*

**A:** We have followed up on these suggestions and revised the manuscript to include more details on the implications of our finding in relation to AD pathology. Some of the excerpts from the manuscript that elaborates on the functional and biophysical implications are as follows:

“In agreement with our finding, enhanced resting Ca^2+^ has been observed in astrocytes from an AD model (Kuchibhotla et al., 2009). However, as our contribution, we predict that this change in resting Ca^2+^ is specific to the decrease in Ca^2+^ binding affinity of PMCA pump (Figures 3B &C). A large fraction of the accumulated Ca^2+^ is sequestered into the ER. Not surprisingly, resting ER Ca^2+^ concentration is also higher in the Aβ-PMCA groups. In line with *in vivo* findings that associates this change in resting Ca^2+^ with synchronous and hyperactive astrocytes, the model predicts high amplitude and frequent Ca^2+^ events in Aβ groups. Astrocytic Ca^2+^ concentration is a critical mechanism for the regulation of blood flow in the arterioles. Astrocytic Ca^2+^ level decreases with excess glutamate in the extracellular space to accommodate the increase in neural activity by an increase in blood flow (Mehina et al., 2017). Lowering of intracellular Ca^2+^ may also reduce astrocytic Ca^2+^ transients and feed forward activation of surrounding neurons through glutamate release. In a striking agreement to the model predictions on dysregulated astrocytic Ca^2+^, both the rigidity and dynamic range of vasculature is compromised in animals displaying excessive amyloid deposition (Kimbrough et al., 2015). These finding, together with model predictions, suggest a prominent role for impaired PMCA signaling in the mediation of amyloid pathology.

Along with the plasma membrane Na^+^/Ca^2+^ exchanger, the PMCA has an important role for astrocytic Ca^2+^ homeostasis by extruding cytosolic Ca^2+^. The model predicts alterations in the kinetics and the rate of Ca^2+^ events in astrocytes with dysfunctional PMCA. The decrease in rise time, full-width and decay time leads to slow and long-lasting Ca^2+^ events to cause more activation of *Syt7* whose kinetics are slow but has a high binding affinity for Ca^2+^. This is also the mechanism behind the observed shift in the balance between kiss-and-run and full fusion releases in the Aβ-PMCA groups (Figure 6C & D). Astrocytes are also known to dynamically alter the balance between transient and slow fusions depending on stimulus characteristics, however, the underlying mechanism is not clear (Malarkey and Parpura, 2011). Here we show that the temporal profile of individual Ca^2+^ is a critical determinant of the mode of fusion. Another key feature that emerges from our model is the presence of spontaneous Ca^2+^ events specifically in the Aβ-PMCA groups. Spontaneous Ca^2+^ transients have been observed in astrocytic processes and are increased in astrocytes with Aβ pathology (Rungta et al., 2016). In our study spontaneous activity is particularly associated with the Aβ-PMCA groups. Supporting our predictions, activating mGluRs did not trigger spontaneous activity (Rungta et al., 2016). Aβ is known to affect membrane receptors that can mobilize Ca^2+^ from outside or from the internal stores (Lee et al., 2014). Here we demonstrate a novel role for PMCA in mediating Aβ-induced Ca^2+^ transients in astrocytic compartments. We also argue that an increase in spontaneous activity depletes the availability of docked vesicles that is necessary for stimulus-dependent fast synchronous release through the kiss-and-run mode (Figure 8B).”

***C44.*** *Talking about synchrony on p20. Aside from the ambiguity of how you look at coherence - coherence of what w.r.t. what? – the critical point here is that the only source of variability is the stochastic equation for h in the IP3R model. What happens if you drop it? Can you please repeat your simulations and analysis of synchrony/coherence without noise first?*

**A:** We thank the reviewer for asking this question. We performed new simulations and analyzed Ca^2+^ and release events from astrocytic compartments without noise (Figure below). We find that channel noise due to stochastic opening of the IP_3_Rs is critical for the generation of mGluR-dependent fast and high amplitude Ca^2+^ events/puffs. This is evident from the complete lack of Ca^2+^ event synchrony in the control astrocytic compartment (Fig. A & B). However, the Aβ groups with dysfunctional PMCAs displayed comparatively higher synchrony when the glutamate stimulation rate was high.

The rate of gliotransmission was also much lower in the deterministic model when compared to the astrocyte model with stochastic IP_3_Rs. Gliotransmission in the deterministic model was dominated by spontaneous and slow asynchronous releases. This led to marginal synchrony of release events in the control group at high glutamate stimulation rates. However, in all the Aβ groups, elevated Ca^2+^ signaling triggered synchronous release events (Fig. D, *left*). This is also apparent from the bar graph that displays average synchrony at low, intermediate and high stimulation regimes (Fig. D, *right*).

**Fig. Synchrony of Ca^2+^ and gliotransmitter release events from astrocytic compartments without stochastic IP_3_Rs.**

**(A)** Synchrony of Ca^2+^ events computed at different interevent intervals is very low in the control compartment but is high in the Aβ group with impaired PMCA. **(B)** Ca^2+^ synchrony averaged across all the interevent intervals at different glutamate stimulation rates. **(C)** Synchrony of gliotransmitter release events computed at different interevent intervals is enhanced in astrocytic compartments with Aβ-induced molecular alterations. **(D)** Gliotransmitter release synchrony averaged across all interevent intervals at different glutamate stimulation rates. The bar graphs indicate average synchrony at different stimulus regimes (low: 0.4-1Hz, mid: 2-10, high: 20-100Hz). Error bars indicate mean standard computed by bootstrapping 100 times with a sample size of 1000.

***C45.*** *You tend to confuse Amyloid-beta pathology with AD. The two things are different: AD is an amyloid-beta pathology, but not all amyloid-beta pathologies are AD. Please avoid ambiguity and clarify.*

**A:** We agree with the reviewer and have revised the manuscript to avoid confusion.

***C46.*** *You describe the increase of resting Ca2+ as a side effect, but it is not. This is a crucial aspect that explains your results related to the alteration of PMCA in terms of calcium signaling and downstream gliotransmitter release in amyloid-related pathology. This also explains why your results in 4K are not surprising.*

A: We have elaborated more on the impact of PMCA-induced shift in resting Ca^2+^ concentration on astrocyte physiology. Below is an excerpt from the manuscript that describes the finding.

“In agreement with our finding, enhanced resting Ca^2+^ has been observed in astrocytes from an AD model (Kuchibhotla et al., 2009). However, as our contribution, we predict that this change in resting Ca^2+^ is specific to the decrease in Ca^2+^ binding affinity of PMCA pump (Figures 3B &C). A large fraction of the accumulated Ca^2+^ is sequestered into the ER. Not surprisingly, resting ER Ca^2+^ concentration is also higher in the Aβ-PMCA groups. In line with *in vivo* findings that associates this change in resting Ca^2+^ with synchronous and hyperactive astrocytes, the model predicts high amplitude and frequent Ca^2+^ events in Aβ groups. Astrocytic Ca^2+^ concentration is a critical mechanism for the regulation of blood flow in the arterioles. Astrocytic Ca^2+^ level decreases with excess glutamate in the extracellular space to accommodate the increase in neural activity by an increase in blood flow (Mehina et al., 2017). Lowering of intracellular Ca^2+^ may also reduce astrocytic Ca^2+^ transients and feed forward activation of surrounding neurons through glutamate release. In a striking agreement to the model predictions on dysregulated astrocytic Ca^2+^, both the rigidity and dynamic range of vasculature is compromised in animals displaying excessive amyloid deposition (Kimbrough et al., 2015). These finding, together with model predictions, suggest a prominent role for impaired PMCA signaling in the mediation of amyloid pathology.”

***C47****. You often say that you apply DHPG to your model. But there is no explanation of how this is achieved in your simulations. You only mention glutamate. I assume that you are just applying a step stimulus, and you imagine that it is equivalent to DHPG in Marchaland et al.’ s experiments. If so, please state it, and avoid ambiguities. Also, you should bear in mind that mGluR affinity changes between glutamate and DHPG.*

**A:** We apologize that we missed out on including the details of how DHPG is simulated in the previous version. We factored in the difference in binding affinity between glutamate and DHPG by updating the *k_a_* (DHPG: 6 µM, glutamate: 11 µM) values in the Hill equation for mGluR-mediated IP_3_ production. We have included this information in the revised manuscript (S1 Table).

***C48.*** *There is a problem with your figures. First, I do not understand whether it is a platform issue, or you have uploaded the figures as I have them effectively, but their resolution is low. They seem to have been uploaded as PNG. This is not the ideal format: you should provide them in PDF or EPS. If you are going for PNG, then provide them with a resolution of at least 1200x1200. The ones that I have are barely readable.*

**A:** We have uploaded all figures of the revised manuscript in EPS format.

***C49****. Have you contacted the authors to re-use their data from Ref. 12? How did you obtain those data? I do not see any credits in the manuscript.*

**A:** We would like to clarify the reviewer that we didn’t use figures from published manuscripts in this study. All the figures are our own. We only used published data which to our understanding is not subject to copyright. We have duly acknowledged for all the data that we gathered from published studies.

***C50****. p23: “To examine this link deeper… and release events in AD astrocytes is a direct outcome of the rapid depletion of docked vesicles” – not clear where and how you see it.*

**A.** We observed a nonlinear dependency in both peak cross-correlation and percentage of docked vesicles with stimulation frequency (Fig. 8A & B). This led us to hypothesize the direct link between peak cross-correlation and population of docked vesicles. We have clarified this in the revised manuscript.

**Figures**

***C51****. Figure 1: Instead of using bar plots for your simulated data, use points. Also, plot error bars accordingly. This will apply for panels D–F. The legend should appear in the first plot (D). You may re-arrange in a matrix of 2x3 instead of 3x2.*

**A**: Panels D-G of Figure 1 in the revised manuscript has simulation data plotted as points with error bars, instead of bar plots. The panels have also been rearranged.

***C52.*** *Figure 2: The kinetic schemes should be included in the appendix when you present the model equations for the Syts. A and B panels should be complemented by sample simulations for the two Syts’ kinetics to clarify how they work in the model. Be consistent with your notation: In the text, you use SytX, but in the figure you use Syt-X – perhaps move 3G-H here?*

**A:** We have included the kinetic schemes of synaptotagmins (*Syt4* & *Syt7*) as a separate panel in the appendix (Figure S3). As suggested by the reviewer, we have added new panels to highlight the difference in kinetics and Ca^2+^ binding affinity between *Syt4* and *Syt7* (Figure 2C-H). We have updated the panel (Figure 2A) to be consistent with the notation used in the manuscript.

***C53.*** *Figure 3: Panel B: Slope is misleading: I assume you mean “coefficient.” Panels B – C: where are the error bars on your data points? What do data points vs. dashed lines represent? Panel D, provide axes, please, and plot the raster image in full size as the other panels. Avoid Blue v. Red as they are rendered identically in grayscale. Panels E and F: same considerations as for 1 D–F.*

**A.** We have modified Figure 3 in order to incorporate the reviewer’s suggestions. We have included error bars with all the simulation data (Figure 3B-F).

***C54.*** *Figure 4: Panels A, B, and J, K: What do data points vs. dashed lines represent? Panels A, B: where are the error bars? Panel B: Why two sets of vertical lines? And why negative PMCA current? You can simplify the y-axis scale, using uM/s units instead, and reporting the absolute PMCA-mediated flux. Panels C – E: where are the error bars? Please, specify bar labels in the xaxis for all plot, instead of having labels inside the bars. You can also consider lumping the three histograms into a single bar-plot differentiating among the three variables. Panels F – I: are you using the same stimulus/seed for the random generator for the same colors across different panels? Otherwise, the results are not technically, same colors, but completely different simulations.*

**A:** We have modified all the panels in figure 4 to incorporate the suggestions by the reviewer. Error bars are now included with all the simulation data. The panel with PMCA flux has been modified to display the flux as positive. The unit for PMCA flux has also been changed to uM/s. The stimulus seed for the random generator are different across runs, however, all the trials are plotted in grey.

***C55.*** *Figure 5: Panels A – D: same considerations of 4F – I. Also, why are you considering so many traces here and not as many as in the previous figures? Please try to be consistent. Panels E and F: where are the error bars? Panels G and H: How are you computing the FWHM and the decay time constant? There is no explanation in your methods.*

**A:** We have tried to be consistent in the number of traces (30 trials/group). All the panels with simulation data have error bars (as grey lines). We have included an explanation in the Data Analysis section on how the different metrics of Ca^2+^ events are computed. Below is the excerpt from the manuscript that describes this.

“*Computation of calcium event features*

We computed the calcium event features (peak amplitude, rise time, fullwidth at half-maximum and decay time) by first identifying individual Ca^2+^ event peaks from the continuous trace. A sample data point was marked as peak if its neighboring points are below the set threshold value (300 nM). This was followed by identification of peak widths. Full width at half-maximum was calculated as the horizontal distance between two data points on either side of the peak that are at half the amplitude of the peak height. Rise time was calculated in a similar way as the horizontal distance between two data points before the peak that are at 20% and 80% of the peak height. Whereas, for decay time, the horizontal distance was computed between peak and the data point at 36.8% of the peak. We used the ‘find_peaks’ and ‘peak_widths’ functions in Scipy’s signal module to extract these metrics.”

***C56.*** *Figure 6: Use a 2x 4/3 matrix instead of a 4x 2/1 configuration. Panels A – D: please provide them with axes. I do not understand what the gray bar on the bottom is.*

**A:** We have modified the figure as per the reviewer’s suggestion.

***C57.*** *Figure 7: Polar plots in Panels A and C are hardly readable and informative. I would advise dropping them or provide them as full histograms from 0 to 360 degrees. I would keep only Panels B and C, and lump them along with Panels 8E – G in a new Figure 7. Alternatively, since there is no reason why you want to show only the phase difference for 0.2 Hz rather than for the whole frequency spectrum, you may provide heatmaps in terms of phi vs. nu. Panels B and D: where are the error bars? No need to repeat the legend in these two plots.*

**A:** We have dropped the polar plots of coherence. As suggested by the reviewer we have included full heat maps of synchrony measured for events at different interevent intervals. We have also included error bars in the averaged line plot for synchrony in the control and Aβ groups.

***C58.*** *Figure 8: Panels A – D can be dropped: they are hardly informative. Also, your scale choice for the color bar makes your results scarcely interesting. You may try with a log scale. I would drop this analysis. Panels E – G: where are the error bars?*

**A:** Considering the suggestions by the reviewer, we have updated the panels in figure 8. We have also included error bars in all the panels.

**Discussion**

***C59****. Your discussion is lacking structure, possibly because you failed initially to clarify your research question. Moreover, you fail to put your results in perspective, mostly restating the result rather than elaborating on the functional/pathological implications. A typical example is on p24 when you state that “one of the most important insights (actually predictions) of this study is the observed loss of temporal precision of individual Ca2+ (events) and vesicle release in AD-like astrocytes” Ok, but why is this important for the pathology? Please elaborate.*

**A:** We have thoroughly revised the discussion section which now details elaborately on the biophysical and functional implications of the model predictions.

***C60****. On p25, you mention something about the “increased presence of ER,” but there is nothing in your analysis related to changes in the ER. Or am I missing something?*

**A:** In our astrocyte compartment model we used relatively high ER-to-cytosol volume ratio (0.5). This is in line with the prominent presence of ER tubules in astrocytic processes as reported in the study by Marchaland et. al. More details of cytosol and ER volume and area are in the appendix (S1 Table).

***C61.*** *The last paragraph: “We hypothesize that this mismatch in information transfer at a crucial hippocampal tripartite synapse may contribute to higher-level cognitive deficits associated with AD.” This requires further elaboration. It does not mean anything. Please provide a reference, and make the reader understand how and why.*

**A:** We have removed this sentence in the revised manuscript.

**Grammar**

***C62.*** *Please be careful: many times, you use “through”/“from”/” via” instead of “by;” and “for” instead of “of.”*

**A:** We thank the reviewer for pointing us to these grammatical mistakes. We have corrected these errors in the revised manuscript.

***C63.*** *There are also ambiguous terms: “low endogenous Ca2+ buffering:” low what?*

**A:** We have removed this confusing sentence from the revised version.

***C64****. The first paragraph of “Gliotransmitter release” needs rephrasing.*

**A:** We have modified this paragraph in the revised manuscript.

***C65.*** *… mobile vesicles that are ‘not’ localized in close confined spaces … also, I do not understand the need for quotes around ‘not.’ There is a typo in Syt7 in the following sentence.*

**A:** We thank the reviewer for pointing out these typos. We have corrected these errors in the revised manuscript.

***C66.*** *p14: the first sentence of “Modeling vesicular release at a single astrocytic process” can be dropped: i.e. “Despite several… not present.” No need for a new line after the first paragraph.*

**A:** We have dropped this sentence from the revised manuscript.

***C67****. p15: “perfectly match” → match; arise→ originate; discrete→ distinct?*

**A:** We thank the reviewer for the suggestions. We have updated the revised manuscript accordingly.

***C68****. p17: two critical regulators of astrocytic calcium signaling changed by AD.*

**A:** We thank the reviewer for the suggestion and manuscript has been updated accordingly.

***C69.*** *Lastly → finally.*

**A:** We thank the reviewer for the suggestion and manuscript has been updated accordingly.

***C70.*** *p21: “difficulty”: you mean the current technological limits? You should provide a reference at the end of this sentence. “Apart from … Ca2+ release events” is hard to understand: “While both” → “Although our results are in agreement with experimental studies…” What do you mean by “temporal relationship”?*

**A:** We thank the reviewer for pointing us to this confusion. We have removed this sentence from the revised manuscript.

***C71.*** *p22: All AD groups displayed lower values than control – lower values of what?*

**A:** We thank the reviewer for suggestion. We have removed this sentence from the revised manuscript.

***C72.*** *p23: To address this gap → to fill in the gap… for astrocytic calcium signaling and the downstream gliotransmitter release…*

**A:** We have updated the manuscript with the reviewer’s suggestion.

***C73.*** *p26: underscores → “likely underpins”. Go astray → this is lost; Ab-induced alterations with astrocytic Ca2+ → alterations of what? Drop “at a single process level”. “abnormal Ca2+” where is this evidence?*

**A:** We thank the reviewer for the suggestions. We have updated the manuscript accordingly.

**Reviewer #2**

***C1.*** *Pillai and Nadkarni develop a computational model to link Ca2+ signaling in the processes of astrocytes with the gliotransmitter release by these cells. The model has two main components: (1) Ca2+ dynamics in astrocytes and (2) gliotransmitter release. A rise in Ca2+ concentration in the processes of astrocytes leads to transmitter release through a fast release pathway (termed kiss-and-run mechanism) and slow release pathway. The model reproduces key observations about Ca2+ signals and distribution of transmitter release events. The model is then used to explore the effect of changes in two Ca2+ pathways observed on in Alzheimer’s disease on Ca2+ dynamics. These include reduced plasma membrane Ca2+ ATPase (PMCA) and gain-of-function enhancement of metabotropic glutamate receptors (mGluRs). The model provides some useful insights into how these two pathways affect Ca2+ signaling and gliotransmitter release in Alzheimer’s disease-affected astrocytes.*

*In my view the main contribution of this study is the development of gliotranmistter release model. Overall, I find the study very interesting as it is addressing an important topic. However, there are some key issues that needs to be addressed before accepting the paper for publication.*

**A:** We would like to thank the reviewer for finding our study interesting. Please find

appended below our point-by-point response to the specific questions raised in the review.

***C2****. Page 5: In the Introduction section, the authors state “While extensive experimental data and computational studies exist on somatic Ca2+ dynamics in astrocytes [26,27], a quantitative formulation of Ca2+ dynamics at a single astrocytic process and its relationship to Ca2+ mediated kiss-and-run and full fusion exocytosis has not been described”. I find this statement a little tricky. The second part of the statement is true, i.e. “a quantitative formulation of Ca2+ dynamics at a single astrocytic process and its relationship to Ca2+ mediated kiss-and-run and full fusion exocytosis has not been described”. However, the first part of the statement makes it sounds like the authors are doing something completely different with the Ca2+ dynamics to model Ca2+ signaling in the process. The fact is, both the “somatic Ca2+ dynamics in astrocytes” models developed before and the model presented in this paper are point models and none pay attention to the spatial structure. Thus the notion that their Ca2+ model is a radical departure from the previous models is technically not correct.*

**A**: Our intention was to refer the reader to the single astrocytic process specific details, like ER to cytosol ratio, number of IP3Rs etc. that we have incorporated in the model. However, we agree with the reviewer that it is still a point model and therefore, it is not a drastic departure from the previous versions. We thank the reviewer for pointing this out. We have revised sentence in the new version of the manuscript.

**C3**. *Page 6: Before equation 1 “….therefore on included in the present model” should be “….therefore not included in the present model”*

**A**: We thank the reviewer for spotting this typo. We have corrected the text in the revised manuscript.

***C4.*** *Page 8-10: There is a disconnect between the description of Ca2+ model and gliotransmitter model. A clear description is needed explaining how the kinetic scheme in Figure 2B is related to the release and recycling scheme. For example, how do the “Docked vesicles” or “Release vesicles” relate to the scheme “S0 <->S1 <->S2”? Same applies to Syt7 scheme.*

**A:** We agree with the reviewer and have included two new figures in the revised version of the manuscript to make the relationship between Ca^2+^ signaling and gliotransmission clearer. Moreover, there is a detailed flow chart in the main manuscript (Figure 2B) that elaborates further on the details of the vesicle recycling and gliotransmission (Figure 2A). The supporting figure (S3 Figure) explains the kinetic schemes of both *Syt4* and *Syt7*. Finally, additional information on *Syt4* and *Syt7* is included in the appendix (S3 Appendix).

***C5.*** *Similarly, what does the statement “We characterized the release machinery by clamping intracellular Ca2+ levels and measuring peak release (R) rate, which was fitted to a Hill equation (4) with Vmax, Kd and coeff as free parameters” mean for the model? And how is equation (4) connected to the gliotransmitter model?*

**A:** We fitted peak release rates with a Hill equation to extract binding affinity and calcium cooperativity of the two synaptotagmins. We have added extra figures in the revised manuscript that plots both the fits and the simulation data (Figure 2C-H). Equation 4 is based on previous works on calcium cooperativity of presynaptic release, first introduced by Dodge and Rahamimoff (Dodge and Rahamimoff, 1967). Here we used a similar approach to characterise and predict the behavior of the gliotransmitter release model. We believe that this data will be useful to compare the model with experimental data in the future. We have also modified this description for clarity in the new version of the manuscript.

“We characterized the release machinery by clamping intracellular Ca^2+^ levels and measuring peak release (R) rate, which was fitted to a Hill equation (4) with *Vmax*, *Kd,* and *coeff* as free parameters. This fit was used to characterize the dynamics and sensitivity of the Ca^2+^-mediated release machinery. Similar characterizations have been done with neuronal synaptotagmins by experimental studies (Dodge and Rahamimoff, 1967; Sun et al., 2007). We believe that this information is vital for comparing the results of the model with future experimental work on astrocytic release machinery.”

***C6.*** *How were the rate equations and parameters in the gliotransmitter model optimized? Were they optimized using trial and error method using the full model (i.e. Ca2+ and gliotransmitter dynamics together)? Or some kind of automated optimization method employing modular approach was used?*

**A:** The parameters of the gliotransmitter release model is largely based on published experimental data (S1 Table). However, we tuned the values slightly to match the release rates with experimental observations. This was done using trial and error methods.

***C7.*** *Page 10-12: I believe the stochasticity in the synchrony between Ca2+ events and release come from the stochastic modeling of gliotranmission. However, this hasn’t been explicitly mentioned in the paper.*

**A:** We thank the reviewer for asking this clarification. We have updated the manuscript to make this clear. Below is the section from the manuscript.

“In order to generate discrete release events at each time step (50 μs), we first computed a probability of release by multiplying the instantaneous release rate from the model with the integration time step. A release event occurred every time the release probability, computed separately for both *Syt4* and *Syt7*, became greater than a uniformly distributed random number between the interval 0 and 1.”

***C8.*** *Page 14, Figure 1: More details about the implementation of DHPG in the model are needed. How is DHPG related to glutamate or mGluR?*

**A:** We implemented DHPG in the model by updating the binding affinity of the mGluR model. We have included this information in the parameter table (S1 Table). We have also updated the section of mGluRs in Appendix (S1 Appendix) to make it clear how we factor in the effect of DHPG in the model.

***C9****. Was glutamate raised to a fixed value for 2 sec or was raised for 2 sec and allow to decay according to first order kinetics? Was the rise a single event or multiple events applied periodically over 2 seconds?*

**A**: We apologize for not including a detailed description of the glutamate stimuli that activated the astrocytic compartment. We have added a separate section (see below) on the revised manuscript that describes the stimulus protocol in more detail.

“We stimulated the astrocytic compartment model with either a single steady-state concentration that lasted the entire duration (2s or 30s) of the stimulus or with repetitive pulses of varying frequencies that resembled presynaptic release. At each release, the glutamate concentration peaked at ~200µM and decayed exponentially with a time constant of 6.25ms (S1 Appendix, S1 Table & S1 Figure). The dynamics of glutamate next to the astrocyte compartment closely followed the time course of glutamate at the perisynaptic region surrounding the astrocytic processes (Matsui, 2005).”

***C10.*** *Page 14, 15, Figure 2B: The statement “Syt4 has a single low-affinity domain (C2B) that binds 2 Ca2+ ions with fast forward rates” is consistent with the kinetic scheme in Figure 2B. However, it is not clear if the mechanism described by the statement “It is also established that Syt4 with a single Ca2+ binding domain can only promote kiss-and-run exocytosis [16]” has been implemented in the model or not. If it is, how? Do states S1 and S2 in the Syt4 scheme both lead to vesicles release? Are the release probabilities in both S1and S2 states the same or different?*

**A:** We thank the reviewer for this important question. In our model, we distinguish between kiss-and-run and full fusion as they follow different time scales of vesicle release, endocytosis, reacidification and docking. Kiss-and-run releases are mediated exclusively through Ca^2+^ binding to *Syt4* which has fast kinetics but low affinity for Ca^2+^. We modelled the activity of *Syt4* using a 3-state kinetic scheme. The state S_0_ is associated with spontaneous release through *Syt4* while Ca^2+^-mediated release is achieved through the S_2_ state (S2 Figure). S_1_ is an intermediary state and does not cause release. We have revised this section for better clarity.

***C11****. Overall, I believe it would make it easier to follow the model if all the rate equations for the gloitransmitter model are listed in a new table.*

A: We agree with the reviewer’s suggestion. All the model equations are now included in the appendix (S1 Appendix) and model parameters are in the accompanying table (S1 Table)

***C12.*** *Page 18 and Figure 5: What was the amplitude and life time (duration for which glutamate was raised) of each glutamate release (spillover)? I believe the amplitude of each individual glutamate release (spillover) event was ~200 uM and was modeled as first order rate equation with a 6.25 ms rise time and 10 ms decay time throughout the paper? If so, this should be explicitly stated on page 9.*

**A:** We thank the reviewer for this question. We have added a figure in the appendix (S1 Figure) that plots the glutamate time course for the model. The dynamics of glutamate is achieved through the glutamate degradation equation (S1 Appendix, section 13). Glutamate is released instantaneously and but decays exponentially with time constant of 6.25ms. We have updated the manuscript with the below text on glutamate dynamics.

“We stimulated the astrocytic compartment model with either a single steady-state concentration that lasted the entire duration (2s or 30s) of the stimulus or with repetitive pulses of varying frequencies that resembled presynaptic release. At each release, the glutamate concentration peaked at ~200µM and decayed exponentially with a time constant of 6.25ms (S1 Appendix, S1 Table & S1 Figure). The dynamics of glutamate next to the astrocyte compartment closely followed the time course of glutamate at the perisynaptic region surrounding the astrocytic processes (Matsui, 2005).”

***C13.*** *Page 20: The reference (kuchibhotla2008) needs to be fixed.*

**A:** We thank the reviewer for spotting this typo in the manuscript. We have corrected this in the revised version.

***C14****. Line 1, page 24: “ER Ca2+ levels (400 mM)” should be “ER Ca2+ levels (400 uM)”?*

**A:** We apologize for this mistake and have corrected it in the revised version.

**Reviewer #3**

Reproducibility report.

**A:** We have provided detailed instructions for running the simulation and analysis to reproduce the figures in the manuscript in GitHub (https://github.com/anupgp/astron.git).

**Reviewer #4**

***C1.*** *This work is a model study of cellular mechanisms associated with Alzheimer's disease.*

*Elucidation of the causes of excessive accumulation of beta-amyloid and the mechanisms of cleansing brain tissue is currently a hot topic in brain physiology. At the same time, another important part in the area is to establish the cellular mechanisms that accompany the accumulation of beta-amyloids. On this basis, we can conclude that the problem solved in this work is of interest as a small puzzle piece that contributes to a general understanding of the problems associated with Alzheimer's disease. It is valuable for the specialists in the field, while might be less understandable for the general public.*

*The manuscript contains a very detailed, even meticulous, description of the modeled cellular pathways and the rationale for the choice of parameters. This allows me to agree with the authors' assertion that they "presented a detailed biophysical model" of the processes under consideration. According to my knowledge, this is the first model to quantitatively predict the release of gliotransmitter in response to beta-amyloid accumulation.*

**A:** We thank the reviewer for the comments and very encouraging remarks on our study.

***C2****. From the results obtained, I would single out two. The first is the answer to the question of what shifts in calcium dynamics are caused by the changes characteristic of the accumulation of beta amyloid. On a qualitative level, this answer could be obtained using a simpler model. The second result is a aberration of the causal relationship (worsening correlation) by bursts of calcium and the release of gliotransmitter.*

**A:** We thank the reviewer for finding the predictions from our study relevant for understanding the impact of amyloid beta on astrocytic Ca^2+^ signaling and gliotransmitter release.

***C3.*** *In line with the above, my assessment of the work presented is positive. I have no serious comments, and I think that the work can be accepted for publication.*

**A:** We thank the reviewer for accepting our manuscript for publication.

***C4****. The discussion of the discussion is quite lengthy, and in the absence of short Conclusions is difficult to read. I would suggest either split the discussion into subsections with subtitles, or add conclusions with a really short summary.*

**A:** We thank the reviewer for the important suggestion. We have completely revised the discussion section to make it concise. We have also added a short summary of the results at the beginning of discussion. We have also added a separate paragraph on the conclusions of the study at the end of the manuscript. Hope that these and the other revisions have greatly enhanced the readability of the manuscript.

**References**

Araque A, Carmignoto G, Haydon PG, Oliet SHR, Robitaille R, Volterra A (2014) Gliotransmitters travel in time and space. Neuron 81:728–739.

Bazargani N, Attwell D (2016) Astrocyte calcium signaling: The third wave. Nat Neurosci 19:182–189.

Bowser DN, Khakh BS (2007) Two forms of single-vesicle astrocyte exocytosis imaged with total internal reflection fluorescence microscopy. Proc Natl Acad Sci U S A 104:4212–4217.

Buskila Y, Bellot-Saez A, Morley JW (2019) Generating Brain Waves, the Power of Astrocytes. Front Neurosci.

Calì C, Bezzi P (2010) CXCR4-mediated glutamate exocytosis from astrocytes. J Neuroimmunol.

Carvalho-Paulo D, de Morais Magalhães NG, de Almeida Miranda D, Diniz DG, Henrique EP, Moraes IAM, Pereira PDC, de Melo MAD, de Lima CM, de Oliveira MA, Guerreiro-Diniz C, Sherry DF, Diniz CWP (2018) Hippocampal astrocytes in migrating and wintering semipalmated Sandpiper calidris pusilla. Front Neuroanat.

Clements J, Lester R, Tong G, Jahr C, Westbrook G (1992) The time course of glutamate in the synaptic cleft. Science (80- ) 258:1498–1501.

Crippa D, Schenk U, Francolini M, Rosa P, Verderio C, Zonta M, Pozzan T, Matteoli M, Carmignoto G (2006) Synaptobrevin2-expressing vesicles in rat astrocytes: Insights into molecular characterization, dynamics and exocytosis. J Physiol 570:567–582.

De Pittà M, Goldberg M, Volman V, Berry H, Ben-Jacob E (2009) Glutamate regulation of calcium and IP3 oscillating and pulsating dynamics in astrocytes. J Biol Phys.

Di Castro MA, Chuquet J, Liaudet N, Bhaukaurally K, Santello M, Bouvier D, Tiret P, Volterra A (2011) Local Ca2+ detection and modulation of synaptic release by astrocytes. Nat Neurosci 14:1276–1284 Available at: http://www.nature.com/doifinder/10.1038/nn.2929.

Diamond JS (2005) Deriving the glutamate clearance time course from transporter currents in CA1 hippocampal astrocytes: Transmitter uptake gets faster during development. J Neurosci.

Dodge FA, Rahamimoff R (1967) Co‐operative action of calcium ions in transmitter release at the neuromuscular junction. J Physiol 193:419–432.

Foley J, Blutstein T, Lee S, Erneux C, Halassa MM, Haydon P (2017) Astrocytic IP3/Ca2+ signaling modulates theta rhythm and REM sleep. Front Neural Circuits.

Hestrin S, Galarreta M (2005) Synchronous versus asynchronous transmitter release: A tale of two types of inhibitory neurons. Nat Neurosci.

Hires SA, Zhu Y, Tsien RY (2008) Optical measurement of synaptic glutamate spillover and reuptake by linker optimized glutamate-sensitive fluorescent reporters. Proc Natl Acad Sci U S A.

Jourdain P, Bergersen LH, Bhaukaurally K, Bezzi P, Santello M, Domercq M, Matute C, Tonello F, Gundersen V, Volterra A (2007) Glutamate exocytosis from astrocytes controls synaptic strength. Nat Neurosci 10:331–339.

Kimbrough IF, Robel S, Roberson ED, Sontheimer H (2015) Vascular amyloidosis impairs the gliovascular unit in a mouse model of Alzheimer’s disease. Brain.

Kuchibhotla K V, Lattarulo CR, Hyman BT, Bacskai BJ (2009) Synchronous hyperactivity and intercellular calcium waves in astrocytes in Alzheimer mice. Science 323:1211–1215.

Lee L, Kosuri P, Arancio O (2014) Picomolar amyloid-β peptides enhance spontaneous astrocyte calcium transients. J Alzheimer’s Dis 38:49–62.

Liou J, Kim ML, Won DH, Jones JT, Myers JW, Ferrell JE, Meyer T (2005) STIM is a Ca2+ sensor essential for Ca2+-store- depletion-triggered Ca2+ influx. Curr Biol.

Liu T, Sun L, Xiong Y, Shang S, Guo N, Teng S, Wang Y, Liu B, Wang C, Wang L, Zheng L, Zhang CX, Han W, Zhou Z (2011) Calcium Triggers Exocytosis from Two Types of Organelles in a Single Astrocyte. J Neurosci 31:10593–10601 Available at: http://www.jneurosci.org/cgi/doi/10.1523/JNEUROSCI.6401-10.2011.

Malarkey EB, Parpura V (2011) Temporal characteristics of vesicular fusion in astrocytes: Examination of synaptobrevin 2-laden vesicles at single vesicle resolution. J Physiol 589:4271–4300.

Marchaland J, Cali C, Voglmaier SM, Li H, Regazzi R, Edwards RH, Bezzi P (2008) Fast Subplasma Membrane Ca2+ Transients Control Exo-Endocytosis of Synaptic-Like Microvesicles in Astrocytes. J Neurosci 28:9122–9132.

Matrosov V, Gordleeva S, Boldyreva N, Ben-Jacob E, Kazantsev V, De Pittà M (2019) Emergence of Regular and Complex Calcium Oscillations by Inositol 1,4,5-Trisphosphate Signaling in Astrocytes. In: Computational Glioscience, pp 151–176.

Matsui K (2005) High-Concentration Rapid Transients of Glutamate Mediate Neural-Glial Communication via Ectopic Release. J Neurosci 25:7538–7547 Available at: http://www.jneurosci.org/cgi/doi/10.1523/JNEUROSCI.1927-05.2005.

Mehina EMF, Murphy-Royal C, Gordon GR (2017) Steady-state free Ca2+ in astrocytes is decreased by experience and impacts arteriole tone. J Neurosci.

Panatier A, Vallée J, Haber M, Murai KK, Lacaille JC, Robitaille R (2011) Astrocytes are endogenous regulators of basal transmission at central synapses. Cell 146:785–798.

Parpura V, Grubišic V, Verkhratsky A (2011) Ca2+ sources for the exocytotic release of glutamate from astrocytes. Biochim Biophys Acta - Mol Cell Res 1813:984–991.

Parpura V, Haydon PG (2000) Physiological astrocytic calcium levels stimulate glutamate release to modulate adjacent neurons. Proc Natl Acad Sci 97:8629–8634 Available at: http://www.pnas.org/cgi/doi/10.1073/pnas.97.15.8629.

Perea G (2005) Properties of Synaptically Evoked Astrocyte Calcium Signal Reveal Synaptic Information Processing by Astrocytes. J Neurosci 25:2192–2203 Available at: http://www.jneurosci.org/cgi/doi/10.1523/JNEUROSCI.3965-04.2005.

Potokar M, Kreft M, Lee SY, Takano H, Haydon PG, Zorec R (2009) Trafficking of astrocytic vesicles in hippocampal slices. Biochem Biophys Res Commun.

Potokar M, Kreft M, Pangršič T, Zorec R (2005) Vesicle mobility studied in cultured astrocytes. Biochem Biophys Res Commun.

Potokar M, Vardjan N, Stenovec M, Gabrijel M, Trkov S, Jorgačevski J, Kreft M, Zorec R (2013) Astrocytic vesicle mobility in health and disease. Int J Mol Sci 14:11238–11258.

Rao SK, Huynh C, Proux-Gillardeaux V, Galli T, Andrews NW (2004) Identification of SNAREs Involved in Synaptotagmin VII-regulated Lysosomal Exocytosis. J Biol Chem 279:20471–20479.

Robinson IM, Ranjan R (2002) Synaptotagmins I and IV promote transmitter release independently of Ca2+binding in the C2A domain. Nature 418:336–340.

Roos J, DiGregorio PJ, Yeromin A V., Ohlsen K, Lioudyno M, Zhang S, Safrina O, Kozak JA, Wagner SL, Cahalan MD, Veliçelebi G, Stauderman KA (2005) STIM1, an essential and conserved component of store-operated Ca 2+ channel function. J Cell Biol.

Rungta RL, Bernier LP, Dissing-Olesen L, Groten CJ, Ledue JM, Ko R, Drissler S, Macvicar B a. (2016) Ca2+ transients in astrocyte fine processes occur via Ca2+ influx in the adult mouse hippocampus. Glia.

Santello M, Bezzi P, Volterra A (2011) TNFα Controls Glutamatergic Gliotransmission in the Hippocampal Dentate Gyrus. Neuron 69:988–1001.

Savtchouk I, Di Castro MA, Ali R, Stubbe H, Luján R, Volterra A (2019) Circuit-specific control of the medial entorhinal inputs to the dentate gyrus by atypical presynaptic NMDARs activated by astrocytes. Proc Natl Acad Sci U S A.

Savtchouk I, Volterra A (2018) Gliotransmission: Beyond black-and-white. J Neurosci.

Srinivasan R, Huang BS, Venugopal S, Johnston AD, Chai H, Zeng H, Golshani P, Khakh BS (2015a) Ca2+ signaling in astrocytes from Ip3r2−/− mice in brain slices and during startle responses in vivo. Nat Neurosci 18:708–717.

Srinivasan R, Huang BS, Venugopal S, Johnston AD, Chai H, Zeng H, Golshani P, Khakh BS (2015b) Ca2+ signaling in astrocytes from Ip3r2−/− mice in brain slices and during startle responses in vivo. Nat Neurosci 18:708–717 Available at: http://www.nature.com/doifinder/10.1038/nn.4001.

Stenovec M, Kreft M, Grilc S, Potokar M, Kreft ME, Pangršič T, Zorec R (2007) Ca2+-dependent mobility of vesicles capturing anti-VGLUT1 antibodies. Exp Cell Res.

Sun J, Pang ZP, Qin D, Fahim AT, Adachi R, Südhof TC (2007) A dual-Ca2+-sensor model for neurotransmitter release in a central synapse. Nature 450:676–682.

Tang W, Szokol K, Jensen V, Enger R, Trivedi CA, Hvalby Ø, Johannes Helm P, Looger LL, Sprengel R, Nagelhus EA (2015) Stimulation-evoked Ca2+ signals in astrocytic processes at hippocampal CA3-CA1 synapses of adult mice are modulated by glutamate and ATP. J Neurosci 35:3016–3021.

Thomas DM, Ferguson GD, Herschman HR, Elferink L a (1999) Functional and biochemical analysis of the C2 domains of synaptotagmin IV. Mol Biol Cell 10:2285–2295.

Vecchio F, Lizio R, Frisoni GB, Ferri R, Rodriguez G, Babiloni C (2011) Electroencephalographic rhythms in alzheimer’s disease. Int J Alzheimers Dis.

Verkhratsky A, Rodríguez-Arellano JJ, Parpura V, Zorec R (2017) Astroglial calcium signalling in Alzheimer’s disease. Biochem Biophys Res Commun.

Vig M, Peinelt C, Beck A, Koomoa DL, Rabah D, Koblan-Huberson M, Kraft S, Turner H, Fleig A, Penner R, Kinet JP (2006) CRACM1 is a plasma membrane protein essential for store-operated Ca 2+ entry. Science (80- ).

Vincent AJ, Gasperini R, Foa L, Small DH (2010) Astrocytes in Alzheimer’s disease: Emerging roles in calcium dysregulation and synaptic plasticity. J Alzheimer’s Dis 22:699–714.

Volterra A, Liaudet N, Savtchouk I (2014) Astrocyte Ca2+ signalling: An unexpected complexity. Nat Rev Neurosci 15:327–335 Available at: http://dx.doi.org/10.1038/nrn3725.

Wallach G, Lallouette J, Herzog N, De Pittà M, Jacob E Ben, Berry H, Hanein Y (2014) Glutamate Mediated Astrocytic Filtering of Neuronal Activity. PLoS Comput Biol 10.

Wolfes AC, Ahmed S, Awasthi A, Stahlberg MA, Rajput A, Magruder DS, Bonn S, Dean C (2017) A novel method for culturing stellate astrocytes reveals spatially distinct Ca2+ signaling and vesicle recycling in astrocytic processes. J Gen Physiol 149:149–170 Available at: http://www.ncbi.nlm.nih.gov/pubmed/27908976%5Cnhttp://www.jgp.org/lookup/doi/10.1085/jgp.201611607.

Wong MY, Cavolo SL, Levitan ES (2015) Synaptic neuropeptide release by dynamin-dependent partial release from circulating vesicles. Mol Biol Cell.

Zhang Q, Fukuda M, Van Bockstaele E, Pascual O, Haydon PG (2004) Synaptotagmin IV regulates glial glutamate release. Proc Natl Acad Sci 101:9441–9446 Available at: http://www.pnas.org/cgi/doi/10.1073/pnas.0401960101.

Zorec R, Araque A, Carmignoto G, Haydon PG, Verkhratsky A, Parpura V (2012) Astroglial excitability and gliotransmission: an appraisal of Ca2+ as a signalling route. ASN Neuro 4:103–119.
