# Supplementary material for: Amyloid pathology disrupts gliotransmitter release in astrocytes
Source: PLoS Comput Biol. 2022 Aug 1;18(8):e1010334. doi: 10.1371/journal.pcbi.1010334 (PMC9371304; doi:10.1371/journal.pcbi.1010334)
Supplement: Attachment — Submitted filename: Pillai_et_al_2022_response_to reviewers.docx [file pcbi.1010334.s013.docx]

**Responses to Reviewer 1**

*Title:*

*Your title is emblematic of my above remarks. "Amyloid pathology disrupts gliotransmitter release in astrocytes" is arguable on many levels. First, "Amyloid pathology" does not mean anything. Amyloids can be several, and you are considering only beta-amyloids. Pathology is not necessarily associated with amyloids. "Disruption" is imprecise since it implies deviation from a norm that is not clear. "Gliotransmitter release in astrocytes" is almost tautological, besides the fact that it should be gliotransmitter release from astrocytes. I would frankly advise editing the title along the lines of something more specific of your study: "Amyloid-beta decorrelate calcium dynamics from gliotransmission in astrocytes."*

**Response:** We appreciate the reviewer’s suggestion and considered it seriously. We also sought the opinion of several experts from across the world about this suggestion from the reviewer. All of them agreed with our view that ‘Amyloid pathology’ clearly indicates pathological manifestations of amyloids. The rest of the title, “ … disrupts gliotransmitter release in astrocytes,” is self-evident and indicates a deviation from the norm, exactly what our results show and what we intend to say. We also intentionally chose “ … *in* astrocytes” instead of ‘*by’*  as the pathological manifestation is ‘*in*’ the astrocyte gliotransmitter release molecular pathway. Therefore, we decided to keep the title as is (Amyloid pathology disrupts gliotransmitter release in astrocytes). An excellent example to illustrate our point is the title of a recent book on the subject, “Computational Glioscience (Springer Series in Computational Neuroscience) 1st ed. 2019, by Maurizio De Pittà (Editor), Hugues Berry (Editor)”. The term “Glioscience” does not exist in the dictionary yet. However, not only is it perfectly acceptable to the scientific community, it tells you precisely what you will find in the book.

• *The title page in the Acknowledgements: "and by the Indian... and Research at Pune."*

**Response:** We thank the reviewer for suggesting the correction. However, we would like to bring to the reviewer’s notice that our institute’s name is ‘Indian Institute of Science Education and Research Pune’. This can also be checked through the website (<https://www.iiserpune.ac.in/>). We, therefore, would like to keep the institution’s name as it is in the ‘Acknowledgement’ section.

**Acknowledgments:** AGP was supported by a Postdoctoral Fellowship from the Indian Institute of Science Education and Research Pune (IISER-P/Ext/PDRF/AP-20145047/10/2016) and Wellcome Trust/DBT India. SN was funded by Wellcome Trust/DBT India Alliance (IA/I/12/1/500529) and by IISER-P.

*Abstract:*

*I would advise rephrasing along the following lines: Extracellular deposition of amyloid-beta (A\beta) is recognized to correlate with synaptic dysfunction and alteration of astrocytic signaling, but the biophysical mechanisms underpinning such alteration remain elusive. On the other hand, astrocytes are known to be active modulators of synaptic function through the regulated release of neuroactive molecules (or gliotransmitters) in a calcium-dependent fashion. We introduce a biophysical model for astrocytic perisynaptic domains to characterize gliotransmission release by A\beta-dependent modulation of astrocytic calcium dynamics through plasma-membrane pumps (PMCA) and metabotropic glutamate receptors (mGluRs). Our model realistically captures astrocytic hyperactivity in the presence of A\beta-related pathologies. At the same time, it also predicts faster rates of gliotransmitter depletion that ultimately decorrelate gliotransmitter release from underpinning calcium dynamics, with the possibility to disrupt the delicate control of synaptic function by astrocytes.*

**Response:** We appreciate the suggestion. Keeping in mind the essence of the feedback, we have revised the abstract.

*Authors' Summary*

*The first sentence is incorrect. Again: the fact that astrocytic signaling is critical for information processing at synapses is not proven; it is just speculated. "Aberration" is not the right word. The signaling that you consider – gliotransmission – has only little been linked with AD. The further reference to technological limitations comes out of the blue since you don't address this aspect in your work, and it is also not the main point of your study. Conclusions: rewrite it as a simplified version of the Abstract just for the lay public.*

**Response:** We appreciate the suggestion. However, we believe that the reviewer is mistaken in their critique. Other scientists in the field and we have provided substantial evidence of the critical role played by astrocytes for a variety of tasks, including information processing. The evidence appears in several review articles and book chapters. Here is one recent example; Astrocyte function from information processing to cognition and cognitive impairment, Mirko Santello, Nicolas Toni & Andrea Volterra; *Nature Neuroscience* **volume 22**, pages154–166 (2019)

***Introduction***

*• There are three main themes in your work: Amyloid-beta, astrocytic calcium signaling, and gliotransmission. You are linking Abeta with calcium signaling and calcium signaling with gliotransmission, but what I am missing to close the logical loop is why we should care about gliotransmission in the context of amyloid-beta—mediated pathologies. You do not provide a structured, logical flow to account for these three factors. This is not only at the Introduction level but also in the presentation of the model, exposition of Results, and arguably in the Discussion. It should not be that difficult: it is only a matter of linking three concepts. The way you currently present your thoughts is that because healthy synaptic function relies on gliotransmission, which is not a sound argument, then Alzheimer's disease, which involves synaptic dysfunction, could be caused by gliotransmission. You see that the cause-effect logic is not consistent. It would help if you based your rationale on a sound syllogism*

**Response:** We appreciate the suggestion. We paid particular attention to the reviewer’s remark on the linking of Aβ with gliotransmission and calcium signaling and modified the ‘Introduction’ appropriately.

*• A related issue is that A\beta links with Alzheimer's disease, and you often interchangeably use A\beta and AD. I would advise focusing your work on A\beta (as I suggested in the Abstract above) and then motivating your interest in A\beta instead by its underpinnings of Alzheimer's disease. This perspective could also offer you the chance to elaborate your Discussion better, emphasizing the translational implications of your work.*

**Response:** We thank the reviewer for the suggestion. We have avoided interchanging between Aβ and AD as this is potentially confusing for the reader.

*• Based on such considerations, I would reshape Introduction, starting from the general notion of neuronglial interactions at synaptic loci along the following lines:*

**Response:** Considering the reviewer’s suggestion, we have modified the Introduction, Results, and Discussion sections to better align the reader to the major themes of the study.

*• Healthy brain function relies on an intricate interaction between neurons and glial cells at synapses [REF]. <Explain how and the gliotransmitter hypothesis. In doing so, talk about gliotransmission, and then emphasize calcium signaling as the crucial mediator of the phenomenon> (should mention Marchaland's work since this is an essential paper in your work).*

**Response:** We now have an extensive description of Marchaland et al. in the Introduction and have also emphasized the role of astrocytic Ca2+ signaling as the crucial mechanism for astrocyte-neuron feedback.

*• < Then use calcium signaling and its pathological behavior – describe 'pathological' – based on Kuchiblota et al.'s study, to link to pathology and A\beta, with emphasis on the extracellular compartment>.<Finally, elaborate A\beta in the context of AD, and motivate your study accordingly.>*

**Response:** We appreciate the suggestion and have made the appropriate modifications to the Introduction.

*• <You should also include a paragraph that describes state of the art on models of gliotransmission and the computational need for your model>*

**Response:** We have included a paragraph on the state-of-the-art computational models that examined astrocytic Ca2+ signaling, gliotransmission, and Aβ mechanisms.

• *In your current version: Alzheimer's disease (AD) is a highly debilitating prominent neurodegenerative ... [1]. According to the amyloid-beta cascade hypothesis, one of the most prevalent neuro-centric theories for AD-related dementia, amyloid-beta accumulation in the extracellular space is the pathological hallmark of AD, and synaptic dysfunction [2]< Note here that synaptic dysfunction is another concept. Your reasoning has a gap.> However, Recent... [5,6]< Panatier did not support this argument; Savtchouck's work is on NMDARs related to gliotransmission... these are not wrong references.> It is now widely accepted (maybe YOU are widely accepting, not me or others) that optimal< who ever spoke even of optimality principles?> interaction between neurons and astrocytes is crucial for normal brain functioning [7-11]< Only ref. 8 could be pertinent here, yet it is not even the most appropriate since it deals with gliotransmission only. You need more general work: probably Khakh and Sofroniew NRN 2016, or maybe the Kastaneka et al.'s roadmap paper in Glia 2016.>*

**Response:** We appreciate the suggestion. As mentioned before, we have modified the Introduction appropriately.

*•... In vivo observations of elevated ... In this study, we focus on two crucial calcium signaling mechanisms... Apart from elevated ... However there is little understanding... The whole logical flow here is broken—no clear cause-effect relation. Different concepts are coming into place. Lousy.*

**Response:** We appreciate the time taken by the reviewer to share their detailed personal opinion on how our manuscript is written. We have also spent an enormous amount of time addressing the reviewer’s concerns. However, we believe using a word like ‘lousy’ in this highly respected professional forum is unacceptable. We have repeatedly seen this tone throughout this review process and are deeply disturbed by it. We want to ask would the same tone or words would be used if the authors were from the USA or Europe or all male or all white. Both authors of the manuscript under consideration have several years of experience writing papers that have been received well by the scientific community. We have never come across a review as condescending and demeaning in tone as this one. We have also reviewed papers for several journals (including PLOS Computational Biology), been careful with our words, and treated our colleagues with respect.

*•... In high levels on astrocytic compartments... Moreover, we consider < use list>: (i) the stochastic gating of IP3Rs; and (ii) different molecular mechanisms for gliotransmission, respectively by kissand-run vs. full-fusion of gliotransmitter-containing vesicles [11, 25 Replace by De Pitta et al., Neurosci. 2016].*

**Response:** We added reference to the study by De Pitta et al. and have modified the sentence to make it more understandable.

*• Drop "Taking into account these molecular... [8,9,11,23]*

**Response:** As per the reviewer’s suggestion, we have dropped the sentence in the new version.

**Methods**

*• What I found disturbing, to say the least, is that you talk about spatially-related quantities "IP3R clusters," "vesicle movement," or "trafficking," and so on, when your model does not include space at all. These terms would make sense only if you were dealing with PDE models. But you are just dealing with lumped compartments. Avoid such ambiguity, please.*

**Response:** The model does not take space into account. This is pretty clear from the manuscript, as mentioned by the reviewer. The use of the term “clusters” in our model refers to numbers. Further, movement refers to changes in time. However, in order to avoid any confusion that some readers might have, we have avoided referring to our description of IP3Rs as a cluster.

*• The whole introductory part of Methods is not informative unless you describe the essence of your model by listing all the variables as: dC_cyt/dt = f_1(c,I,G...) dI/dt = f_2(C_cyt, I, h...) dS/dt = ... ... Otherwise, drop it. Likewise, I would drop, whenever possible, details on parameter choices (as these are detailed in the supplementary tables).*

**Response:** We have removed the introductory part in the methods section and refrained from mentioning the parameters in the main text. They are now detailed in the appendices.

*• Moreover, what is this use of uncommon abbreviations like "conc:" what do you think you are doing? Writing a shopping list?*

**Response**: The use of the abbreviation conc. appears in several instances in the literature. Here is a random instance where you will find this appearing (Agarwal et al. Neuron 2017). A simple search for the term 'conc.' in Pubmed leads us to over a hundred pages of references. Surely, these papers are not all shopping lists. However, for better clarity, we have belabored the point and used the full-term concentration instead. Again, we find the reviewer's tone completely unacceptable and lacking in professionalism.

*Your notation also is not consistent. Sometimes you use K_D, other times k_d, other times K_d. Please be coherent.*

**Response:** We thank the reviewer for pinpointing this problem. We have refrained from using multiple notations when referring to dissociation constant (*k_d_*).

• "... cluster of 5 IP3Rs:" you see? You cannot have a cluster in a lumped parameter model.

**Response:** We understand that the use of the word ‘cluster’ can be misleading to some readers. We have avoided the usage of the word cluster to mean the small number of IP_3_Rs.

*• "The model reliably captured stochastic Ca2+ transient through the IP3R cluster using a Langevin approximation of the Li-Rinzel IP3R model developed by Shuai et al. [43]." What is "reliably" for you? There is no mention of the IP3R cluster in Shuai's original work unless you go to the PDE version of that model. It is not a Langevin approximation (as I pointed out previously). The LR model for IP3R is not correct. Li and Rinzel provided a reduction of the DYK model. Please be careful with these details.*

**Response for incorrect use of the term *Langevin approximation*:** Langevin-like methods for simulating ion-channel gating are used for their computational efficiency to approximate the simulation of Markov chain models. However, the accuracy of Langevin methods to reproduce results of Markovian methods needs to be validated (Accurate Langevin approaches to simulate Markovian channel dynamics, Yandong Huang *et al.* 2015 *Phys. Biol.* **12** 061001). Our usage of "Langevin approximation…” clearly indicates the approximation involved when using Langevin equations for ion-channel gating. We have used the Langevin method for the Li-Rinzel model for IP3 receptors developed and rigorously validated *by Shuai et al.* (Langevin Modeling of Intracellular Calcium Dynamics, ISBN : 978-3-540-40236-7, J.W. Shuai, P. Jung).

We, therefore, disagree with the reviewer's viewpoint. Based on the above references, the term Langevin approximation is unambiguous. However, this argument is futile, pedantic, and takes away from the main focus of the work. We have therefore refrained from using the phrase in the present version.

**Response for** “**There is no mention of the IP3R cluster in Shuai's original work unless you go to the PDE version of that model”:** We want to point out that the reviewer is mistaken here. We quote from the abstract of Shuai et al. (Langevin Modeling of Intracellular Calcium Dynamics, ISBN : 978-3-540-40236-7, J.W. Shuai, P. Jung), "Langevin equation has the same form as the deterministic 2-variable Li-Rinzel model except that a noise-term is added where the strength is related to the size of the release **cluster**."

**Response for “The LR model for IP3R is not correct. Li and Rinzel provided a reduction of the DYK model.”**: We agree that the Li-Rinzel model is a reduced model of the original model proposed by GW De Young and J Keizer. We should also state that there are several instances where this model has been referred to independently, without referring to the history of its development. This is common practice. For example, every reference to the Hindmarsh-Rose or the Fitzhugh-Nagumo model is not preceded by an attempt to trace its roots to the Hodgkin-Huxley model or perhaps the Van der Pol oscillator. The reviewer may have a personal preference for referring to the history of any computational model that they may refer to in their article. We prefer not to.

*“The model includes realistic descriptions of Ca^2+^ buffers and pumps, both within the cytoplasm and ER, as well as a stochastic version of IP_3_Rs that together generates physiologically realistic Ca^2+^ events.”*

*• "... A passive... is not fully understood [not relevant, but if you keep it then a REF is needed].*

**Response:** We have removed this sentence from the recent version.

*• Glutamate stimulation... [44] is a wrong reference.*

**Response:** We thank the reviewer and have corrected this mistake. The updated version of the concerning section is below.

*“Glutamate evoked Ca^2+^ events and gliotransmission at a single astrocytic compartment was examined by stimulating with either steady state pulses (2s & 30s) or by a profile of glutamate as observed at the perisynaptic region close to astrocytic compartments (Bergles and Jahr, 1997; Dzubay and Jahr, 1999; Diamond, 2005). The latter was accomplished using a simple first-order reaction equation that captured both the sharp peak (~200µM) and decay (6.25ms) of glutamate concentration (S1 Appendix, S1 Table & S1 Figure A).”*

*• Eq. 4: Please use conventional symbols: V_max, \kappa_d, coeff-->n. And correct the main text accordingly,*

**Response:** We thank the reviewer for the suggestion. We now refer to these parameters in Hill equations as *V_max_*, *k_d_,* and *n* everywhere in the main text.

*• Eqs. 6 and 9: you are using the same symbol "r" for different things.*

**Response:** We thank the reviewer for spotting this mistake. We have updated these notations to avoid any chance of confusion.

*• The responsive measure r was computed by ... T_0 and T_stim (use the same case for quantities that bear the same dimension).*

**Response:** This issue has been addressed in the revised version.

*• Flip eq. 8 with eq. 9. Move "Asynchrony value of 1 corresponds to..." after equation 9.*

**Response:** The equations have been updated in the new version.

*• "Synchrony was computed for each Ca2+ interevent interval by a matrix of event timings vs. trials following the treatment originally introduced by Pinsky and Rinzel [56]. At each trial j... from now on to eq. 7 whatever you wrote is unreadable.*

**Response:** We have rewritten this section to improve readability.

*• Computation of calcium event features. Full width at half maximum (FWHM) as the time interval between left and right halves of a calcium peak. For rise times, we considered the time interval between... Why do you keep using "horizontal distance"?!? Geese, say things for what they are.*

**Response:** We have updated this section to better explain our method for measuring FWHM, rise time and decay time.

*• C++ (and not C^{++})*

**Response:** The notation for C++ has been updated.

*• we compiled by GCC7.5 (welcome: we are now at version 20+).*

**Response:** Our simulations used GCC 7.5. We have verified that the model can successfully run in GCC ([GCC 12.1](https://gcc.gnu.org/gcc-12/) released on 2022-05-06). We would like to add that established research labs continue to use GCC7.5 for a wide variety of reasons.

*• "institution" what Institution? A Hospital? Or perhaps an Academic Institute? Which one?*

**Response:** We meant our host institute. We thank the reviewer for highlighting this mistake. We have modified the sentence to make it clearer.

***Results***

*• Organize your Results in two-three sections: Calcium/gliotransmission model; Ab dependence... and so on.*

**Response:** The results are now organized in three sections as follows.

*A single compartment model for astrocytic Ca^2+^ signaling and gliotransmission*

*Modeling Aβ conditions in astrocytes*

*Aβ modifies astrocytic Ca^2+^ signaling, gliotransmission, and the temporal relationship between them.*

*• The PMCA pumps present on the plasma membrane mediate... within a process mainly arise by stochastic ... The dynamics of these Ca2+ spikes is also ...*

**Response:** The text has been updated.

*• At some point at p15 (if I counted it right...) "Both fast kiss-and-run-like confined releases and slow-spreading... full-fusion in astrocytes." This whole paragraph pertains to modeling methods rather than to results.*

**Response:** We have accommodated this suggestion and have moved the concerned paragraph to the methods section.

*• On p16: "Similar to a previous study [23]" It is still Marchaland et al. Your phrasing does not make any sense.*

**Response:** We have modified this sentence in the new version.

*• On p18, suddenly out of the blue, you introduce PMCA functionality: "Multiple studies have reported..." It looks like you need to start a new section of Results*.

**Response:** We have modified this section.

*• Soon after on p19 you start talking in terms of astrocytic groups with Ab pathology. Please DO NOT. You are not modeling astrocyte groups. You are simulating different conditions. Likewise, do not use terms such as Ab-groups. Stick to meaningful and transparent definitions such as "Ab conditions," "astrocyte domains" in different conditions, and so on...*

**Response:** We have avoided referring to different Aβ conditions as groups.

*• on p19 Refs 68-70 are not in pathological conditions; hence they are not appropriate.*

**Response:** Here we are merely referring to the glutamate concentration profile used in the model for Aβ conditions, which is the same as in the normal scenario. We have clarified this in the manuscript.

*• Abeta pathology enhances gliotransmitter release events: Start directly from "We next describe the impact of..."*

**Response:** We have modified the text accordingly.

*• "Abeta induces frequency-dependent modulation of Ca2+ event synchrony: synchronous discharges... and pathological roles [add REF]." What the hell are you talking about? "Discharges"?!? Discharge is related to electrical quantities, maybe, but not to chemical signals as in astrocytes. Mind your lexicon. Furthermore: synchronicity requires two terms: what are they?*

**Response:** We would like to have it on record that we find the tone and use of the word “hell ” particularly offensive. We ask the reviewer to please stick to professional, meaningful discourse and language.

The word ‘discharge’ is commonly used in sentences to mean ‘put off’ of something, such as payload, consignment, etc. (https://www.merriam-webster.com/dictionary/discharge). Accordingly, it has been extensively used in scientific literature to mean the release of vesicles or other cargo. As an example, we would like to point to the sentence below from page 2, the last paragraph of the article by Marchaland et al. (Marchaland et al., 2008).

“The sequence of images in Figure 1A shows that, under basal conditions, the pHF signal is very low both under TIRFi and EPIi; only a few fluorescent spots are visible, representing vesicles undergoing spontaneous discharge.”

Our calculation of synchrony is described in detail in the methods section.

*• ... p22: There is accumulating evidence that ... Ca2+ and/or gliotransmitter release events boosts... Therefore, we next investigated... we computed temporal synchrony at different interevent intervals (see Methods) to study... (Figure 7A). • p24. We quantified... -> As expected, the cross-correlation between Ca2+ peaks and gliotransmitter release (see Methods) was high... • p25: ... loss of temporal correlation between calcium and release events in different Ab conditions with respect to physiological ones.*

**Response:** We have adapted the text to incorporate these suggestions by the reviewer.

**Discussion**

*• Abeta, astrocytic Ca2+ signaling, and gliotransmission have remained unclear. Here gliotransmission seems disconnected from the other concepts. Hopefully, by the above suggestions, you will be able to rephrase coherently.*

**Response:** We have modified the discussion section to clearly describe the links between Aβ, Ca^2+^ signalling, and gliotransmission.

*• Our results quantitatively describe... Our model can quantitatively reproduce experimental observations for altered astrocytic calcium spiking statistics mediated in the presence of Ab pathology. Specifically, we pinpoint that such alterations could critically depend on Ab-mediated regulation of mGluR < what> and PMCA <what>*

**Response:** We have elaborated more on Aβ-induced alterations in the dynamics of astrocytic Ca^2+^ events and the role of the two Aβ conditions that were explored in this study.

*• ....We build on previous models of intracellular calcium signaling to seek an accurate description of synaptically-activated gliotransmission at individual perisynaptic astrocytic processes. Our description includes...*

**Response:** We have modified the manuscript to reflect the suggestion by the reviewer.

*• p26 Ca2+ events from astrocytic microdomains... a broad range of kinetics...there is no "kinetics" for Ca2+ signals, rather only "dynamics."*

**Response:** According to the standard definition (<https://www.collinsdictionary.com/dictionary/english/kinetics>) the word kinetics is “another name for ‘dynamics”. In scientific literature, the word kinetics has been regularly used in contexts similar to our usage (‘NMDAR decay kinetics’ in the Abstract of the paper by Myme et al. (Myme, 2003) or to mean the kinetics of quantal events of glutamate in the paper by Matsui et al. (Matsui, 2005). Again, we have used ‘kinetics’ in our manuscript to imply the time course/dynamics of Ca^2+^ events.

To avoid further confusion, we have modified the sentences to make sure that there is no ambiguity in the meaning because of our choice of wording.

*• [30,39,40] these are incorrect. Drop 39 and 40. Add instead Panatier et al., 2011; Bindocci et al., 2017.*

**Response:** We have incorporated this suggestion by the reviewer.

*• p25 "Despite... Ca2+-dependent vesicular release [78,79].” Unrelated. Drop.*

**Response:** This sentence has been dropped.

*• P27 When AD-mediated changes are implemented in our model... > When we considered AD- mediated changes of astrocytic signaling, our model predicts that... consistently with experimental data [13]. Additionally, and in agreement with in vivo findings,."An important prediction of our model is that this change... Ab-PMCA groups" Come on, this is not a prediction. It is evident from your model. Drop it.*

**Response:** We have accommodated this suggestion

*• P28: in striking agreement with the model prediction... what are you talking about? This is speculation. There is no agreement whatsoever. Rephrase.*

**Response:** we have rephrased this section in the revised version of the manuscript.

*• P29: While in vivo studies confirm synchronous Ca2+ activity ... (synchronous w.r.t to what?) ... Evidently, despite the high rate of Ca2+...*

**Response:** The use of the term synchrony in the manuscript refers to the synchrony of Ca^2+^ events across different compartments of astrocytes (Kuchibhotla et al., 2009). Synchrony was examined by computing the cross-correlogram of calcium activity between any two independent trials and was then averaged across all the comparisons. This is described in detail in the methods section.

*• Taken together, it is worth noting that in our simulations, the effect of Abeta is most potent at 0.4-10Hz... so what? Why is it worth noting? What is the logical link with what you told me before?*

**Response:** We have removed this sentence in the revised version.

*C. ... To conclude, we presented a biophysically detailed model to realistically simulate ... to verify our modeling assumptions and provide valuable insights on the role of gliotransmitter release under normal and pathological conditions <again: gliotransmitter release is only marginally discussed in the context of Abeta pathology at this stage>.*

**Response:** We have included an additional paragraph that explains the role of gliotransmission in the context of Aβ conditions.

***Figures***

*• You cite Figure 2 before Figure 1, and several references to Figures are incorrect (e.g., Figure 3 in Discussion is not the right figure).*

**Response:** We have carefully gone over the manuscript to make sure these types of errors are eliminated, and the figures are cited in the right order.

*• Figure 1. A. Since you are considering glutamate spillover, you should sketch some synaptic structures near your synaptic process. B. What are the different colors? Very confusing, frankly. Just show traces in one color. Use dashed lines for DHPG application as elsewhere. D–G Probably, it is better to show these data by histograms rather than piecewise linear curves. F. Use conventional labels: FWHM (ms).*

**Response:** In light of the suggestions by the reviewer, we have made the following changes to the figure.

1. Updated the schematic of the astrocytic compartment to include both pre and postsynaptic structures (Figure 1A).

2. Representative traces now have a uniform color (Figure 1B)

3. Added solid lines to all panels to clearly indicate the time and duration of DHPG application (Figure 1C).

4. All histograms are changed from point/line plots to bar graphs (Figures 1D-1G). Please note that in the first version of the manuscript, the histograms were bar graphs but had to be changed because of the reviewer’s suggestion. For reference, I have pasted below the relevant excerpts from the reviewer’s previous comments.

“*Figure 1: Instead of using bar plots for your simulated data, use points. Also, plot error bars accordingly. This will apply for panels D–F.*”

*• Figure 3. Make D–F plots of the same size of A–C, and vertically align their y-axes to these latter. Here and in the remainder of the figures, they are rendered the same in grayscale if you use blue and red. Use color-blind colors, please: e.g., orange and green/blue and so on. Why not put DHPG on top of all graphs (also in Figure 1) to have the axes start from 0 and improve readability? B and C. Make errorbars thinner and data points larger. D-E, what's the point of showing errorbars/data points if they are within the thickness of the line? Tune line widths in a meaningful fashion, please.*

**Response:** We modified Figure 3 in the following ways as per the reviewer’s suggestion

1. Adjusted size and alignment of all the figure panels.

2. Made sure that all the colors are compatible with color blindness.

3. The solid line indicating DHPG application was moved to the top in all the panels.

4. In all the panels, both x and y axes start near zero

5. All the panels were replotted to make sure that the error bars were clearly visible.

*• Figure 4D. Replot as 2D heatmaps color-coded for calcium concentration. Use the same color scale for all the plots. Alternatively, use the color scheme (after correcting it for blinds) to avoid using titles as x-axes labels. Plot all figures in a standard fashion: no tiny axes vs. full axes: only full axes. Hence show in D trials and time for all panels.*

**Response:** We made several modifications to this figure to incorporate the suggestions by the reviewer.

1. Included representative plots as heat maps instead of plotting actual traces.

2. Use of a color scheme that is adjusted for color blindness.

3. Adjusted all the panels to have all the axes clearly visible

*• Figure 5. A. Same considerations as in Figure 4D. B–F Please add Ticks and Y-label to the right panels. Correct for the color blind.*

**Response:** We made the following modifications to the figure as per the reviewer’s suggestion.

1. Adopted a color scheme that is adjusted for colorblindness

2. All panels have clear tick marks and labels

*• Figure 6A. Drop y-axes between panels except for the leftmost one. But specify ticks and x labels for all panels. Use at least 3-4 x ticks.*

**Response:** We have incorporated the reviewer’s comments in this figure. They are listed below.

1. Instead of having the full Y-axis, only the tick marks and labels were included. However, for all the panels, we included a full X-axis with both ticks and labels.

2. All axes have at least 3 tick marks and labels.

*• Figures 7A,C. Repeat ticks and labels for x and y axes, please. What's this choice of non-equispaced ticks on color bars and x-axes? Change it, please. On the y-axes, also, please adopt ticks with the same number of decimal figures. Up to 4-5 ticks in this case.*

**Response:** Figure 7 has been modified as per the reviewer’s suggestions. The changes are as below.

1. Ticks and labels were included for all X and Y axes. Color bars have equally spaced ticks and markings. Axis labels of all the panels were modified to have the same number of decimal points.

2. All colors have been corrected for color blindness.

**Supplementary Material / Appendix 1**

*• Again, you do not use the consistent notation: Glu is sometimes denoted as a concentration, i.e.
"[Glu]," some other times as a "Glu." Please be consistent.*

**Response:** We now consistently report glutamate concentration as [Glu] in Appendix 1.

*• J_IP3P should have been corrected by now and be instead J_IP5P or J_5P.*

**Response:** We have now mentioned J_IP3P as J_5P_ in the text.

*• Moreover, make sure to number your equations.*

**Response:** We have now added numbers to all the equations in Appendix 1.

*• q_2 should be rather Q_2 for historical reasons*.

**Response:** We thank the reviewer for spotting the typo. We have dropped this term in the new version.

*• Nonconventional symbols for multiplication "x" (again!): please drop them.*

**Response:** We have dropped multiplication symbols from all the equations.

*• What's the point of showing J_syt4/7 if we do not have the equations for S_i / Y_i? Would you please show these equations?*

**Response:** We included equations for all the S_i_ and Y_i_ states in the revised version of the S1 Appendix.

*• The equation for glutamate degradation cannot be written as it is: you need to justify why you do not consider the contribution to degradation by binding by receptors. Also, what is the source of extracellular glutamate? I am missing your "stimulus(t)" equation.*

**Response**. The effective glutamate concentration is modelled using the updated equation below for [Glu](t), which is a stimulus(t) in our case. The source of extracellular glutamate is presynaptic release. These details are now included in the main text in the new version.

[Glu](t) = *Glu_max_*δ(t-t_r_) + [Glu]exp(*k_Glu_*t), t_r_ = glutamate release time

*• Since you deliberately mention Hill functions in your main text, use the classic notation for such functions then: H_n(x, K), to simplify your equations.*

**Response:** We have updated all the Hill equations in Appendix 1 with the simplified notation.
